# Dietary Selenium Deficiency Accelerates the Onset of Aging‐Related Gut Microbial Changes in Aged Telomere‐Humanized Mice, With *Akkermansia muciniphila* Being the Most Prominent and Alleviating Selenium Deficiency‐Induced Type 2 Diabetes

**DOI:** 10.1111/acel.70130

**Published:** 2025-06-20

**Authors:** Ying‐Chen Huang, Hsin‐Yi Lu, Li Zhang, Alicia Olivier, Tung‐Lung Wu, Chuan‐Yu Hsu, Caleb LeGrand, Huawei Zeng, Samantha Curran, Qingzhou Wang, Ramakrishna Nannapaneni, Xue Zhang, Max Ticó, Marco Mariotti, Ryan T. Y. Wu, Gerald F. Combs, Wen‐Hsing Cheng

**Affiliations:** ^1^ Department of Biochemistry, Nutrition, and Health Promotion Mississippi State University Mississippi State Mississippi USA; ^2^ Department of Poultry Science Mississippi State University Mississippi State Mississippi USA; ^3^ Department of Pathobiology and Population Medicine Mississippi State University Mississippi State Mississippi USA; ^4^ Department of Mathematics and Statistics Mississippi State University Mississippi State Mississippi USA; ^5^ Institute for Genomics, Biocomputing, and Biotechnology Mississippi State University Mississippi State Mississippi USA; ^6^ Grand Forks Human Nutrition Research Center USDA Grand Forks North Dakota USA; ^7^ Department of Animal and Dairy Sciences Mississippi State University Mississippi State Mississippi USA; ^8^ Department of Genetics, Microbiology and Statistics Universitat de Barcelona Barcelona Spain; ^9^ Centre for Genomic Regulation The Barcelona Institute for Science and Technology Barcelona Spain; ^10^ Department of Nutrition and Food Science University of Maryland College Park Maryland USA; ^11^ Jean Mayer USDA Human Nutrition Research Center on Aging Tufts University Boston Massachusetts USA; ^12^ Department of Nutrition and Food Sciences Texas Woman's University Denton Texas USA

## Abstract

Previous studies have shown that dietary selenium (Se) deficiency in mice reshapes gut microbiota, exacerbates healthspan deterioration (e.g., type 2 diabetes), and paradoxically activates beneficial longevity pathways. This study demonstrated that dietary Se deficiency accelerated many age‐related gut microbial changes in aged telomere‐humanized C57BL/6J diabetic mice in a sexually dimorphic manner, with *Akkermansia muciniphila* showing the greatest enrichment in males. However, dietary Se deficiency did not enrich *A. muciniphila* in mature or middle‐aged male C57BL/6J wild‐type mice. Oral gavage of *A. muciniphila* alleviated Se deficiency‐induced type 2 diabetes‐like symptoms, reversed mucosal barrier dysfunction and gut inflammation, and resulted in a trend of symbiotic and competitive suppression changes in certain gut bacteria in mature wild‐type mice under conventional conditions. The beneficial effects of *A. muciniphila* appeared to be independent of selenoproteins sensitive to dietary Se deficiency, such as GPX1, SELENOH, and SELENOW, in the liver and muscle. Altogether, these results show that dietary Se deficiency accelerates age‐related *A. muciniphila* enrichment specifically in aged male mice with severe insulin resistance and pancreatic senescence, indicating a potential hormetic response to Se deficiency through reshaped gut microbiota, which alleviates hyperglycemia and partially compensates for healthspan decline.

## Graphical abstract

Gut *Akkermansia muciniphila* is enriched by dietary selenium deficiency in aged telomere‐humanized mice and counteracts dietary selenium deficiency‐induced type 2 diabetes.

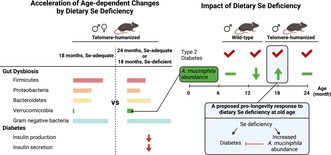

**Abbreviations**
AKT: mouse thymoma viral protooncogene
GPX1: glutathione peroxidase 1
GPX3: glutathione peroxidase 3
SCFA: short‐chain fatty acid
Se: selenium
SELENOH: selenoprotein H
SELENOP: selenoprotein P
SELENOW: selenoprotein W

## 1 Introduction

Selenium (Se), an essential mineral, exerts its physiological functions primarily through selenoproteins. As body Se status decreases, the expression of selenoproteins essential for immediate viability, such as selenophosphate synthetase 2 and glutathione peroxidase 4, is prioritized at the expense of others, such as glutathione peroxidase 1 (GPX1) (Labunskyy et al. 2014; Sunde and Raines 2011). According to the triage theory of aging, chronic Se insufficiency and reduced expression of these “low‐hierarchy selenoproteins” accelerates age‐related degeneration (McCann and Ames 2011). In line with this notion, long‐term dietary Se deficiency renders telomere‐humanized mice susceptible to the early onset of aging (e.g., graying of hair, alopecia, cataracts, and delayed wound healing) and age‐related disorders (e.g., osteoporosis and type 2 diabetes‐like symptoms); however, they also exhibit extended longevity (Wu et al. 2017). This paradox may be reconciled from the perspective of hormesis (Zhang et al. 2018), as dietary Se deficiency in mice is known to activate pro‐longevity pathways (Yim et al. 2019).

Although the composition and abundance of gut microbiota remain relatively stable over time in healthy adults, they shift considerably later in life and are influenced by diet and certain chronic diseases, such as type 2 diabetes (Claesson et al. 2011; Favier et al. 2002; Larsen et al. 2010; Qin et al. 2012; Rampelli et al. 2013). Malnutrition and gut dysbiosis may interact and subsequently promote chronic low‐grade inflammation, contributing to the etiology of type 2 diabetes (Cani and Delzenne 2010). In particular, malnutrition and aging are known to promote dysbiosis of short‐chain fatty acid (SCFA)‐producing bacteria and facultative anaerobes in the gut (Biagi et al. 2012, 2010; Rampelli et al. 2013). According to genome‐wide association data and Mendelian randomization analysis, host genetics predicts a positive association between type 2 diabetes risk and propionate production, but a negative association with butyrate production in the human gut (Sanna et al. 2019).

Evidence suggests competition between gut microbiota and the host for dietary Se, especially when this nutrient is insufficiently available. Germ‐free conditions increase hepatic GPX1 expression in mice fed diets moderately deficient in Se (0.09–0.10 μg Se/kg diet) (Hrdina et al. 2009) or adequate in Se (Kasaikina et al. 2011). Additionally, about a quarter of bacteria require Se to express selenoproteins (Zhang et al. 2006). The health‐promoting roles of dietary Se in gut homeostasis are demonstrated by several observations: gut dysbiosis in growing conventional mice fed a Se‐deficient diet (Kasaikina et al. 2011), increased inflammation and intestinal cancer in *Gpx1* ^−/−^ *Gpx2* ^−/−^ mice (Chu et al. 2004), and improved well‐being in stressed piglets fed Se‐enriched probiotics (Gan et al. 2014).

Because dietary Se deficiency most significantly impacts metabolic pathways in aged telomere‐humanized mice, as supported by the concurrent induction of type 2 diabetes‐like symptoms (Wu et al. 2017), we hypothesized that gut microbial dysbiosis contributes to Se deficiency‐induced type 2 diabetes during aging. Herein, results from aged telomere‐humanized mice showed that long‐term dietary Se deficiency accelerated age‐related gut microbial changes, with an increased abundance of *A. muciniphila* being most prominent, either due to Se deficiency or age (18 vs. 24 months) in males. Next, causal relationships between *A. muciniphila* administration and Se deficiency‐induced diabetes‐like symptoms were assessed. In middle‐aged mice pretreated with antibiotics and in mature conventional male mice, neither group showed *A. muciniphila* enrichment due to Se deficiency. However, *A. muciniphila* oral gavage alleviated type 2 diabetes‐like symptoms in Se‐deficient mice, and this protection was associated with reduced gut inflammation.

## 2 Results

### 2.1 Early Onset of Changes in the Gut Microbiota of Telomere‐Humanized Diabetic Mice Between 18 and 24 Months of Age due to Dietary Se Deficiency

**FIGURE 1:**
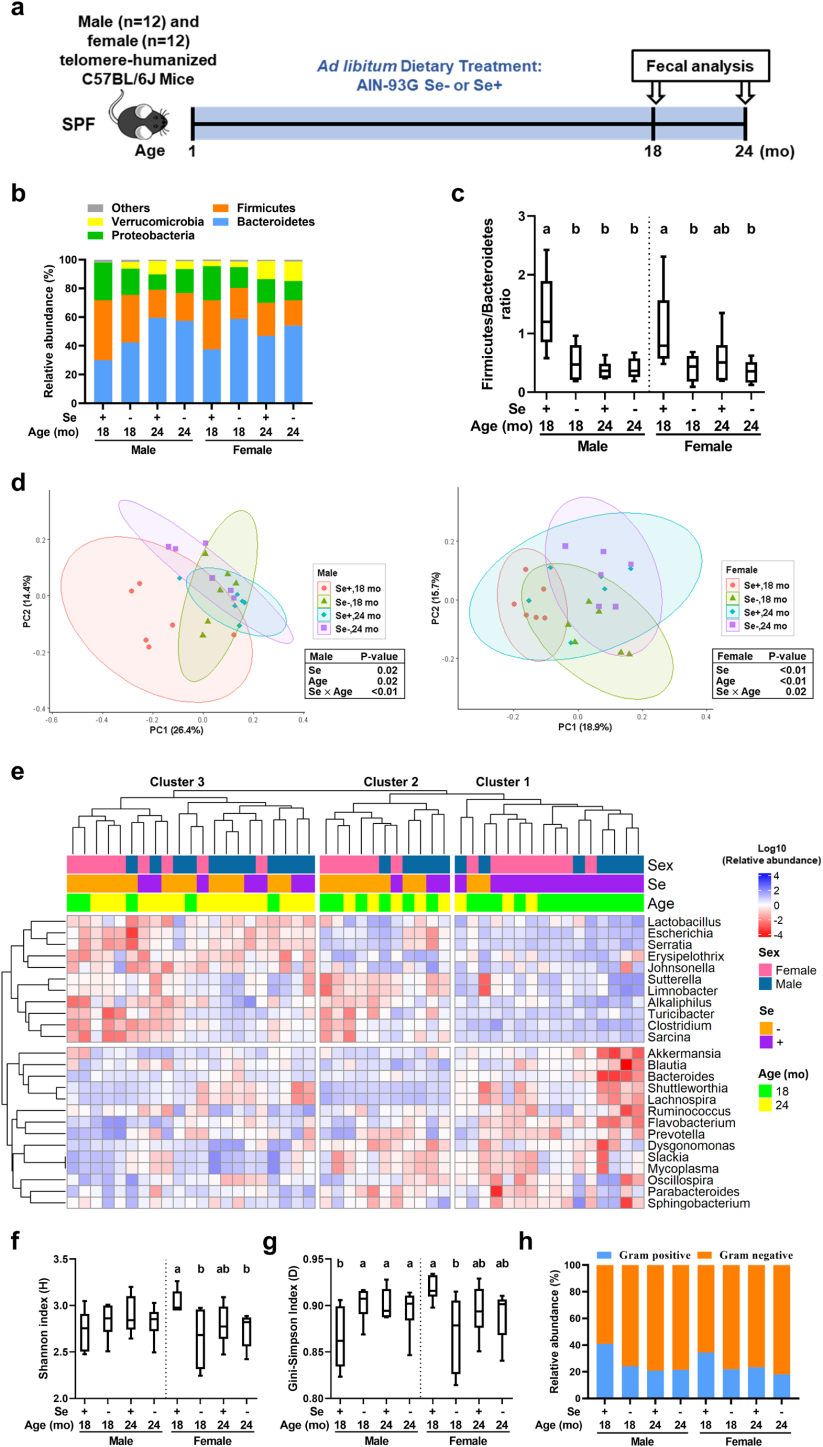
Dietary Se deficiency and a 6‐month increase in age similarly reshape gut bacterial communities in aged telomere‐humanized mice using fecal samples. (a) Study design. (b) Relative abundances of bacterial phyla. (c) Firmicutes/Bacteroidetes ratios. β‐diversity (d) and α‐diversity (Shannon [f] and Gini–Simpson [g] indices) based on Illumina sequencing results at the species level. For β‐diversity, group‐level differences were analyzed using permutational multivariate analysis of variance. (e) Heatmap analysis of the predominant genera. (h) Relative abundances of gram‐positive and gram‐negative bacteria. Box plots show means and 25th to 75th percentiles. Values without sharing a common letter within the same sex differ, *p* ≤ 0.05. *n* = 6 per group. Se+, Se‐adequate diet; Se−, Se‐deficient diet; SPF, specific pathogen‐free condition.

The impact of dietary Se deficiency and aging on the gut microbiota was investigated in telomere‐humanized mice aged 18 and 24 months, which have previously been shown to exhibit early onset of type 2 diabetes‐like symptoms due to dietary Se deficiency (Wu et al. 2017). Sequencing of the V3‐4 region of *16S rRNA* gene amplicons from fecal samples of the same cohort of mice used previously (Figure 1a; Wu et al. 2017) revealed that, consistent with prior studies (Langille et al. 2014; Zhang et al. 2013), 97.7% of the microbial composition at the phylum level was dominated by Bacteroidetes, Firmicutes, Proteobacteria, and Verrucomicrobia (Figure 1b). Compared to 18‐month‐old male Se‐adequate mice, the relative abundance of Bacteroidetes and Verrucomicrobia was increased in those aged 24 months by 1‐fold and 91‐fold, respectively, and enriched by Se deficiency by 40% and 46‐fold, respectively (Figure 1b). Similar trends, though to a lesser extent, were observed in female mice. In contrast, Se deficiency and aging from 18 to 24 months reduced the abundance of Firmicutes and Proteobacteria in both sexes. Compared to 18‐month‐old Se‐adequate mice, the Firmicutes/Bacteroidetes ratio, a marker of aging and inflammation (Mäkivuokko et al. 2010; Sokol et al. 2008), decreased in 24‐month‐old mice and by Se deficiency in both sexes (Figure 1c). Neither Se deficiency nor aging from 18 to 24 months affected total bacterial counts in either sex (Figure S1).

The Bray–Curtis index, calculated using principal component analysis, revealed significant group separation (*p* < 0.05) due to Se deficiency and aging from 18 to 24 months in male and female telomere‐humanized mice. Both clusters were positively shifted along the first principal component (PC1) (Figure 1d), suggesting that Se deficiency and aging similarly impacted gut bacterial composition in the mice. Hierarchical clustering of genus‐level taxa showed that Se‐adequate males and females aged 18 months clustered together, representing 69% of cluster 1 (*p* < 0.05), further corroborating the decoupling of bacterial communities due to Se deficiency or aging from 18 to 24 months (Figure 1e). The composition distribution did not differ (*p* = 0.75) by sex across clusters. Cluster 1 was dominated by taxa such as *Lactobacillus*, *Escherichia*, and *Serratia*, and was sparse in *Akkermansia*, *Blautia*, and *Bacteroidetes*. Nonparametric Shannon (Figure 1f) and Gini‐Simpson (Figure 1g) indices showed that Se deficiency decreased microbial diversity in 18‐month‐old female mice, while it resulted in an earlier onset of the increase in diversity from 18 to 24 months in males, as indicated by the Gini‐Simpson index only. In addition to this sexual dimorphism, bacterial diversity was lower in Se‐adequate males than in females at 18 months. Given the mathematical principles of these indices, the Gini‐Simpson index was more suitable for comparing microbial communities (Nagendra 2002). In males, 41% of the gut microbiota in Se‐adequate 18‐month‐old mice was gram‐positive, but this dropped to 21%–24% with Se deficiency or aging from 18 to 24 months; a similar trend was observed in females (Figure 1h). Overall, dietary Se deficiency reshapes the gut microbiota toward an aged state in both sexes, with sexual dimorphism observed in within‐community variations.

### 2.2 Effects of Dietary Se Deficiency and Aging on the Abundance of Selenoprotein‐Rich, Short‐Chain Fatty Acid‐Producing, and Lactic Acid‐Producing Taxa in Aged Telomere‐Humanized Diabetic Mice

**FIGURE 2:**
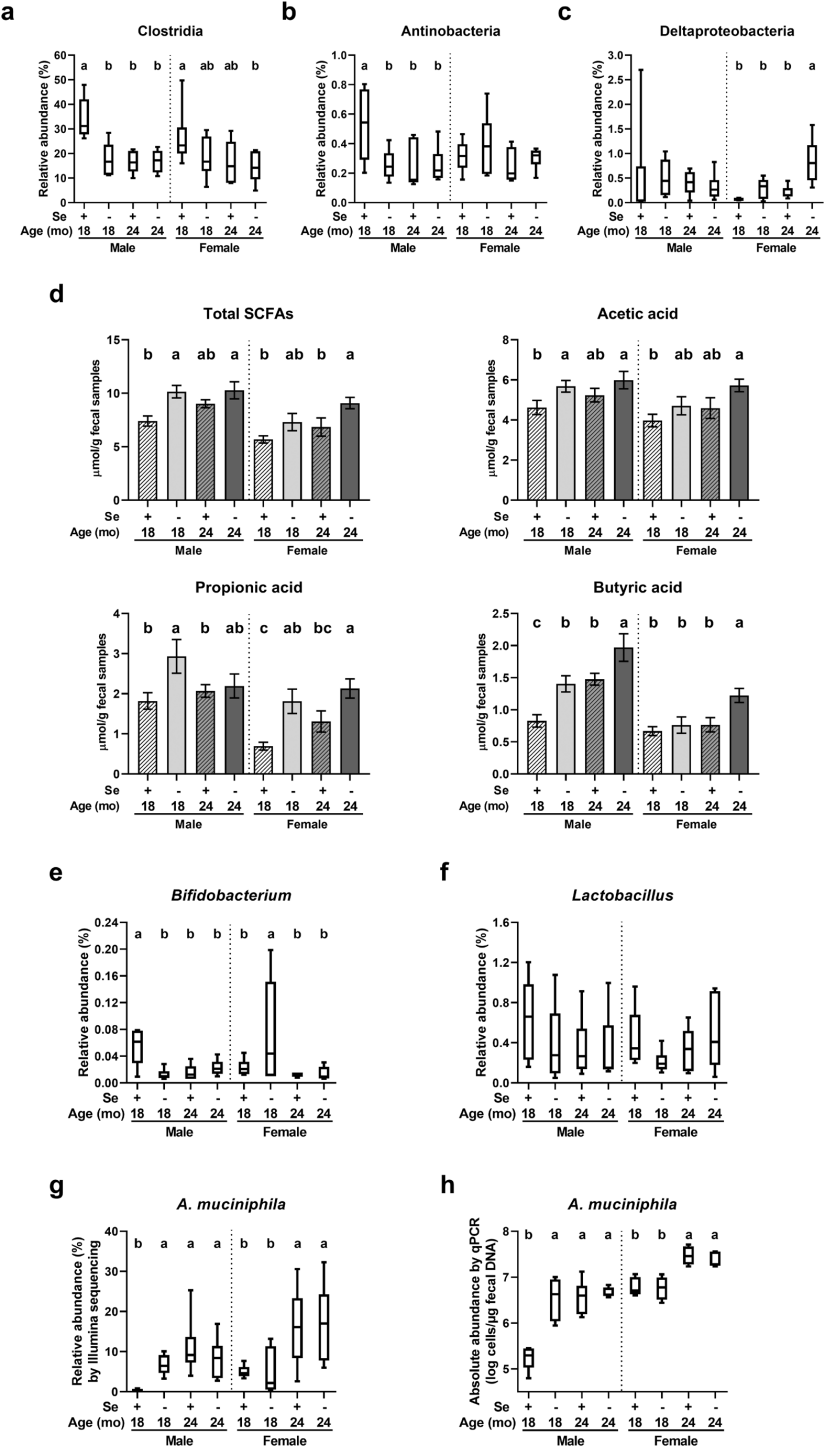
Impacts of dietary Se deficiency and a 6‐month increase in age on the abundances of selective taxa and fecal SCFA in aged telomere‐humanized mice. (a–c) Selenoprotein‐rich class Clostridia and phyla Actinobacteria and Deltaproteobacteria. (d) Fecal SCFA concentrations. (e, f) Lactic acid‐producing genera *Bifidobacterium* and *Lactobacillus*. (g, h) *A. muciniphila*. Abundances were determined by 16S rDNA sequencing results (a–c; e–g) or qPCR (h). Values without sharing a common letter within the same sex differ, *p* ≤ 0.05. SCFA, short‐chain fatty acid; Se+, Se‐adequate diet; Se−, Se‐deficient diet.

Selenoproteins are estimated to be expressed in 21.5% of the 349 sequenced bacteria, primarily in the Clostridia class (anaerobic, gram‐positive) and the phyla Actinobacteria (aerobic, gram‐positive) and Deltaproteobacteria (aerobic, gram‐negative) (Zhang et al. 2006). Compared to Se‐adequate mice aged 18 months, Se deficiency and a 6‐month increase in age similarly decreased the relative abundance of Clostridia and Actinobacteria in males, but had no effect on Deltaproteobacteria in males or on any of the three taxa in female telomere‐humanized mice (Figure 2a–c). In contrast, the relative abundance of Deltaproteobacteria increased in Se‐deficient females with aging from 18 to 24 months and in 24‐month‐old females with Se deficiency, but not in males (Figure 2c). Altogether, dietary Se deficiency results in an earlier onset of aging‐related decreases in these two gram‐positive, selenoprotein‐expressing taxa, predominantly in aged male mice.

Of the 10 SCFA‐producing *Clostridium* clusters IV and XIVa analyzed (Figure S2), compared to Se‐adequate telomere‐humanized mice aged 18 months, Se deficiency increased the relative abundance of *Butyrivibrio* and *Catonella* and decreased that of *Roseburia* in males, but had no effect on any of the taxa in females. A 6‐month increase in age increased the abundance of *Ruminococcus*, *Faecalibacterium*, *Butyrivibrio*, and *Catonella* in males, and *Coprococcus* in females, while decreasing that of *Roseburia* in males. Among these changes, Se deficiency accelerated the aging‐related increases in *Butyrivibrio* and *Catonella* and the decrease in *Roseburia*, but only in males. Next, gas chromatographic analysis showed that Se deficiency increased fecal concentrations of: (1) total SCFAs, acetic acid, propionic acid, and butyric acid in males, and propionic acid in females at 18 months; (2) butyric acid in males, and total SCFAs, propionic acid, and butyric acid in females at 24 months (Figure 2d). Aging from 18 to 24 months increased fecal concentrations of butyric acid in males fed Se‐deficient or Se‐adequate diets and in females fed a Se‐deficient diet, but had no effect on total SCFAs, acetic acid, or propionic acid in either sex. For lactic acid‐producing bacteria, compared to 18‐month‐old Se‐adequate mice, *Bifidobacterium* abundance was reduced by Se deficiency and aging in males and increased by Se deficiency but was not influenced by aging in females (Figure 2e). In contrast, neither Se deficiency nor aging impacted *Lactobacillus* abundance in either sex (Figure 2f). Overall, these results show: (1) a temporal trend of increased fecal concentrations of total SCFAs and butyric acid due to Se deficiency in males before females; and (2) butyric acid as the most prominent SCFA, enriched by Se deficiency or aging in aged telomere‐humanized males.

### 2.3 *A. muciniphila* Is Enriched by Dietary Se Deficiency Only in Aged Male Telomere‐Humanized Mice and Is Predicted Not to Express Selenoproteins or Utilize Se

Among all the taxa analyzed, *Akkermansia muciniphila*, a mucin‐degrading, SCFA‐producing species of Verrucomicrobia (Derrien et al. 2004), showed the greatest increase in relative abundance due to Se deficiency (22‐fold) and a 6‐month age increase (38‐fold) in males, compared to Se‐adequate telomere‐humanized mice aged 18 months. In females, its abundance increased moderately (2.4‐fold) with aging but was unaffected by Se deficiency (Figure 2g). qPCR analysis confirmed these changes (Figure 2h). Overall, *A. muciniphila* appears to be the most prominent fecal bacterium exhibiting Se deficiency‐induced earlier onset of aging‐related changes, with enrichment observed only in aged male telomere‐humanized mice. In contrast, *A. muciniphila* abundance was reduced due to dietary Se deficiency in the feces of middle‐aged (13‐month‐old) and unaffected in the fecal and cecal contents of mature (8‐month‐old) male wild‐type mice (Figure 6d–f).

To investigate whether *A. muciniphila* encodes selenoproteins and other known traits of Se utilization (Manta et al. 2022), relevant gene markers were searched in four *Akkermansia* reference genomes (see Section 4.11 for details). While Se‐independent genes from several bacterial selenoprotein families (e.g., methionine sulfoxide reductase A, peroxiredoxin, and peroxiredoxin‐like protein) were detected, no markers of Se utilization, including selenoproteins, were identified. This suggests that the observed effects of Se deficiency on *A. muciniphila* abundance are indirect, and that it does not compete with the host for dietary Se in the gut.

### 2.4 *A. muciniphila* Alleviates Type 2 Diabetes‐Like Symptoms in Middle‐Aged Wild‐Type Mice Pretreated With Antibiotics and Fed a Se‐Deficient Diet

**FIGURE 3:**
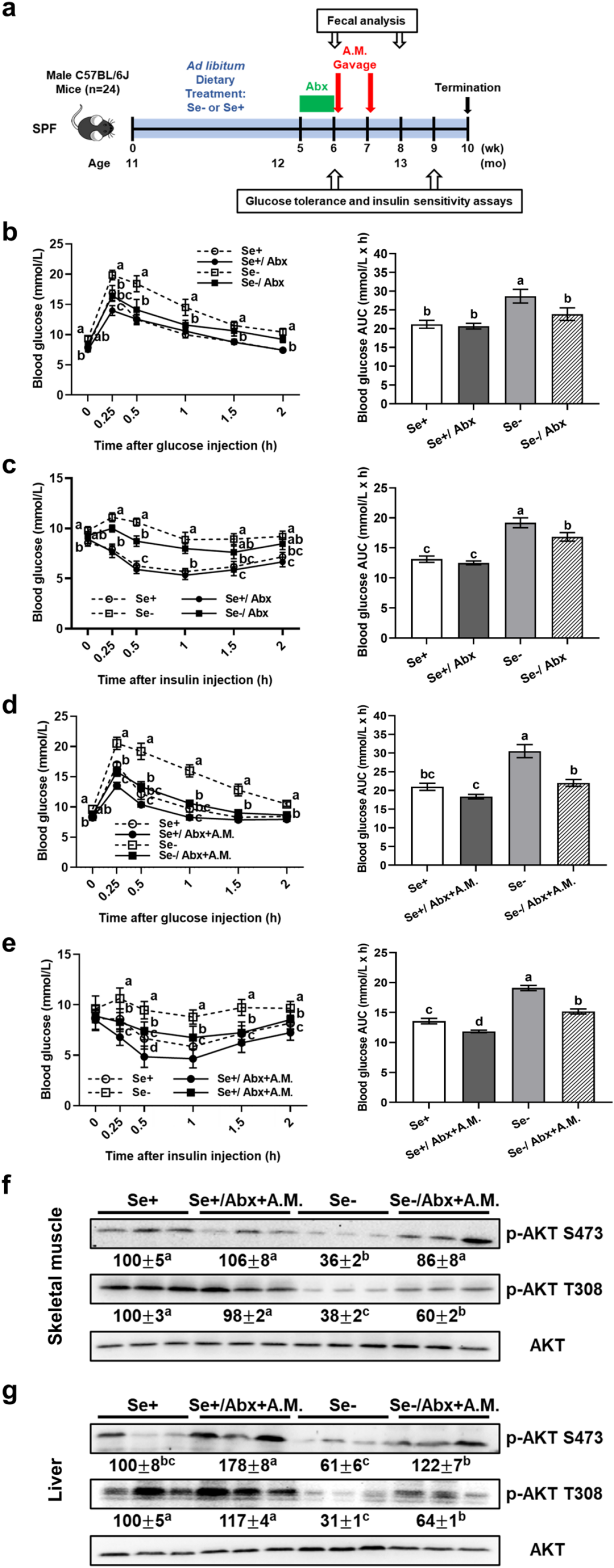
*A. muciniphila* oral gavage alleviates type 2 diabetes‐like symptoms in antibiotic‐pretreated middle‐aged C57BL/6 mice fed a Se‐deficient diet. (a) Study design: antibiotics were treated via drinking water and daily oral gavage; *A. muciniphila* was given weekly by oral gavage at 1 × 10^9^ CFU (detailed in Section 4.1). Glucose tolerance and insulin sensitivity assays were performed after antibiotic pretreatment (week 6; b, c) and with *A. muciniphila* oral gavage (week 9; d, e). Left panels show blood glucose concentrations over time; right panels show AUC. Skeletal muscle (f) and liver (g) AKT phosphorylation were determined by Western analysis (week 10). Values (means ± SEM, *n* = 6) without sharing a common letter differ, *p* ≤ 0.05. Abx, antibiotics; AKT, mouse thymoma viral protooncogene; A.M., *A. muciniphila*; AUC, area under the curve; Se+, Se‐adequate diet; Se−, Se‐deficient diet.

**FIGURE 4:**
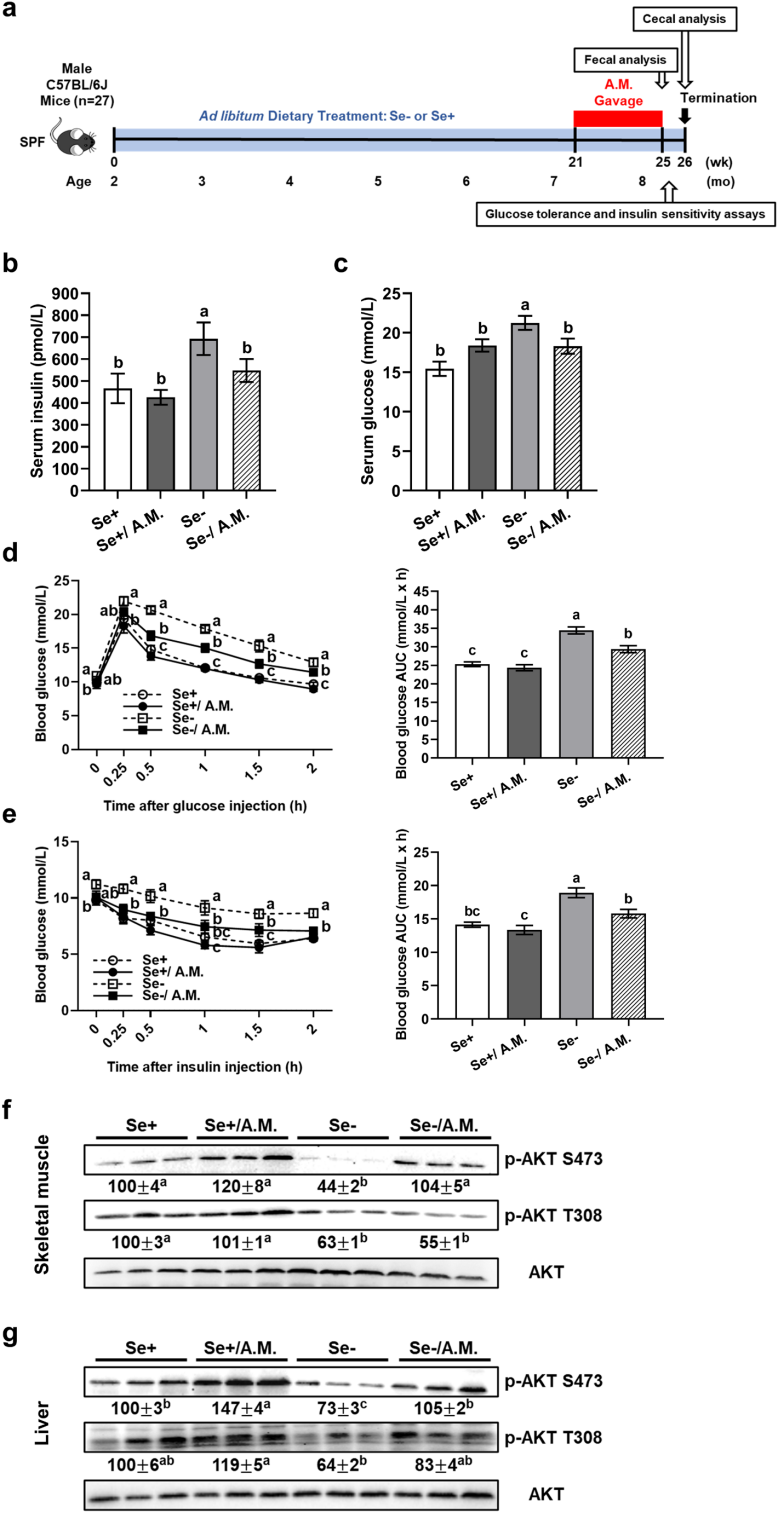
*A. muciniphila* oral gavage alleviates type 2 diabetes‐like symptoms in mature conventional male C57BL/6 mice fed a Se‐deficient diet. (a) Study design. *A. muciniphila* was administered daily by oral gavage at 2 × 10^8^ CFU. (b, c) Postmortem fasting serum insulin and glucose concentrations. (d, e) Glucose tolerance and insulin sensitivity assays after *A. muciniphila* oral gavage (week 25). Left panels show blood glucose concentration over time; right panels show AUC. (f) Skeletal muscle and (g) liver AKT phosphorylation (week 26) were determined by Western analysis. Values (means ± SEM, *n* = 6–7) without sharing a common letter differ, *p* ≤ 0.05. AKT, mouse thymoma viral protooncogene; A.M., *A. muciniphila*; AUC, area under the curve; Se+, Se‐adequate diet; Se‐, Se‐deficient diet.

*A. muciniphila* is known to alleviate type 2 diabetes, as demonstrated in mouse and human studies (Bárcena et al. 2019; Depommier et al. 2019; Everard et al. 2013; Shin et al. 2014). Previous research has linked dietary Se deficiency to type 2 diabetes‐like symptoms in aging‐accelerated telomere‐humanized mice (18–24 months) and wild‐type mice (8–13 months) (Huang et al. 2021; Wu et al. 2017). Therefore, the causal effect of *A. muciniphila* oral gavage on dietary Se deficiency‐induced type 2 diabetes was assessed in antibiotic‐pretreated middle‐aged (Figure 3a) and conventional mature (Figure 4a) male wild‐type mice without intrinsic *A. muciniphila* enrichment (Figure 6d–f).

The antibiotic treatment nearly purged the five bacterial species we tested (Figure S3), enabling studies of the specific roles of live *A. muciniphila* enrichment by limiting confounding influences from other bacteria. Interestingly, the antibiotic treatment improved glucose tolerance (by 17%; Figure 3b) and insulin sensitivity (by 12%; Figure 3c) in Se‐deficient but not Se‐adequate mice, suggesting that dietary Se deficiency reshaped the gut microbiota to a composition favoring type 2 diabetes. *A. muciniphila* oral gavage in the antibiotic‐treated condition enhanced glucose tolerance (Figure 3d) in Se‐deficient (by 28%) but not Se‐adequate mice, as well as insulin sensitivity (Figure 3e) in both Se‐deficient (by 21%) and Se‐adequate (by 13%) mice. Post‐mortem analyses of the insulin signaling pathway revealed that Se deficiency reduced (62%–69%) baseline AKT phosphorylation at the serine residue (p‐AKT S473) and the threonine residue (p‐AKT T308) in skeletal muscle, and p‐AKT T308 in the liver (Figures 3f,g and S4). *A. muciniphila* oral gavage increased (58%–139%) levels of p‐AKT S473 and T308 in muscle and liver of Se‐deficient mice but did not influence those in Se‐adequate mice, except for a 78% increase in liver p‐AKT S473 level. *A. muciniphila* oral gavage increased fecal relative abundance of *A. muciniphila* by 228‐fold in antibiotic‐pretreated mice (Figure 6d). Se deficiency did not influence body weight (Figure S5a) or food intake (Figure S5b) during the entire 10‐week time course, except for food intake fluctuations (i.e., a decrease at week 6 and an increase at week 10) in the mice with mock oral gavage. Although *A. muciniphila* oral gavage did not affect body weight, it increased food intake at weeks 8–10 in Se‐adequate mice. The 1‐week antibiotic treatment reduced body weight in Se‐deficient mice and food intake in Se‐adequate mice. Protein levels of serum SELENOP and GPX3 and skeletal muscle and liver GPX1, SELENOH, and SELENOW (Figure S6a–c) were reduced (26%–76%) by Se deficiency, but the changes were not influenced by *A. muciniphila* oral gavage, suggesting that *A. muciniphila* oral gavage did not alleviate type 2 diabetes‐like symptoms in the Se‐deficient mice through these selenoproteins.

### 2.5 *A. muciniphila* Alleviates Type 2 Diabetes‐Like Symptoms in Mature, Conventional Wild‐Type Mice Fed a Se‐Deficient Diet

Antibiotic pretreatment reduces confounding bacterial effects but induces metabolic changes (Figure 3b,c; Bongers et al. 2022). Moreover, the onset of type 2 diabetes typically occurs in middle age. To corroborate the observed protection by *A. muciniphila* against dietary Se deficiency‐induced type 2 diabetes‐like symptoms, mature wild‐type mice under conventional conditions were employed (Figure 4a). Dietary Se‐deficiency increased fasting insulin (54%; Figure 4b) and glucose (33%; Figure 4c) concentrations in postmortem sera, induced glucose intolerance (38%; Figure 4d) and insulin resistance (31%; Figure 4e), and decreased (27%–56%) baseline levels of skeletal muscle p‐AKT S473 and T308, as well as liver p‐AKT S473 (Figures 4f,g and S7). All these defects in Se‐deficient mice were alleviated by oral gavage of live *A. muciniphila* , except for p‐AKT T308. In Se‐adequate mice, *A. muciniphila* oral gavage only increased liver p‐AKT S473 levels (47%; Figure 4g). Neither dietary Se deficiency nor *A. muciniphila* oral gavage altered fasting serum triglyceride, cholesterol, or HDL concentrations (Figure S8a–c). Dietary Se deficiency reduced (40%–81%) protein levels of serum GPX3, skeletal muscle GPX1, SELENOH, and SELENOW, and liver GPX1 and SELENOW; however, these changes were unaffected by *A. muciniphila* oral gavage (Figure S9a–c). In the mice aged 7–8 months, dietary Se deficiency did not affect body weight or food intake, while *A. muciniphila* oral gavage reduced body weight throughout the entire time course and food intake one week thereafter in Se‐adequate mice (Figure S10a,b). Altogether, these results in mature mice living in a specific‐pathogen‐free environment corroborate those in antibiotic‐pretreated middle‐aged mice.

### 2.6 *A. muciniphila* Alleviates Mucosal Barrier Dysfunction and Inflammation in the Gut of Mature, Conventional Wild‐Type Mice Fed a Se‐Deficient Diet

**FIGURE 5:**
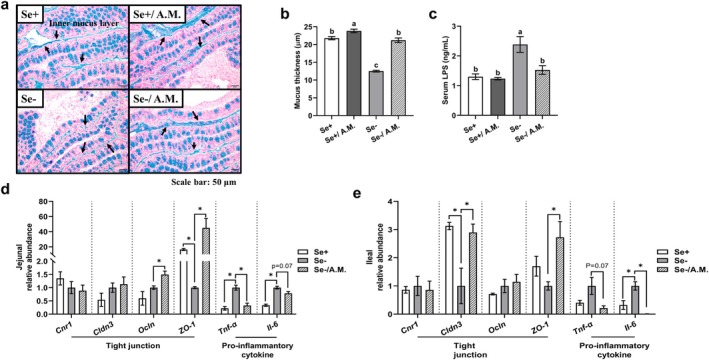
*A. muciniphila* oral gavage alleviates gut barrier dysfunction and inflammation in mature conventional male C57BL/6 mice fed a Se‐deficient diet. (a, b) Mucus layer thickness, representative Alcian blue staining images, and quantification. Arrows indicate the inner mucus layer; blue represents goblet cells; pink represents colon epithelial tissues. (c) Postmortem fasting serum lipopolysaccharide concentration. (d, e) Tight junction and pro‐inflammatory cytokine mRNA expression in the jejunum and ileum based on RT‐qPCR analyses. Values (means ± SEM, *n* = 6–7) without sharing a common letter or with a bracket when compared pairwise differ (*p* ≤ 0.05 or as indicated). *p < 0.05; A.M., *A. muciniphila*; *Cldn3*, claudin 3; *Cnr1*, cannabinoid receptor 1; *Il‐6*, interleukin 6; LPS, lipopolysaccharide; *Ocln*, occludin; Se+, selenium‐adequate diet; Se*−*, selenium‐deficient diet; *Tnf‐α*, tumor necrosis factor α; *ZO‐1*, zonula occludens‐1.

Homeostatic interactions between the gut microbiota and the mucus layer optimize barrier functions (Johansson et al. 2011). Compared to a Se‐adequate diet, dietary Se deficiency reduced mucus layer thickness by 43% in proximal colon segments; however, *A. muciniphila* oral gavage reversed this defect (Figure 5a,b). Consistent with the link between mucosal barrier dysfunction, inflammation, and leaky gut (Camilleri 2019), Se deficiency increased serum lipopolysaccharide concentrations by 84%, but *A. muciniphila* oral gavage reverted the induced inflammation (Figure 5c). RT‐qPCR analysis of other gut barrier markers revealed that Se deficiency reduced mRNA expression levels of *ZO‐1* (zonula occludens‐1) in the jejunum (94%; Figure 5d) and *Cldn3* (claudin 3) in the ileum (68%; Figure 5e); however, *A. muciniphila* oral gavage reversed these impairments with a 43.9‐fold increase in *ZO‐1* and a 1.9‐fold increase in *cldn3*. Additional pairwise comparisons of mRNA levels showed: (1) upregulation by *A. muciniphila* oral gavage of jejunal *Ocln* (occluding) and ileal *ZO‐1* under Se deficiency, and jejunal and ileal *Cldn3*, *Ocln*, and *ZO‐1* under Se adequacy (Figures 5d,e and S11a,b); and (2) downregulation by Se deficiency of ileal *Cldn3* and *ZO‐1* in *A. muciniphila* ‐treated mice (Figure S11d). For pro‐inflammatory cytokines, Se deficiency increased *Tnf‐α* and *Il‐6* mRNA levels in the jejunum and *Il‐6* in the ileum, while *A. muciniphila* oral gavage reduced these levels by 21%–99% (Figure 5d,e). Other mRNA changes included: (1) downregulation of ileal *Tnf‐α* by *A. muciniphila* oral gavage under both Se deficiency and adequacy conditions; and (2) paradoxical downregulation of ileal and upregulation of jejunal *Il‐6* due to Se deficiency in *A. muciniphila* ‐treated mice (Figures 5e and S11b–d). Altogether, *A. muciniphila* oral gavage alleviates leaky gut and associated molecular changes in mature, conventional wild‐type mice fed a Se‐deficient diet.

### 2.7 Trends in Symbiotic and Competitive Suppression Changes in the Gut of Mature and Middle‐Aged Mice Following *A. muciniphila* Oral Gavage and Dietary Se Deficiency

**FIGURE 6:**
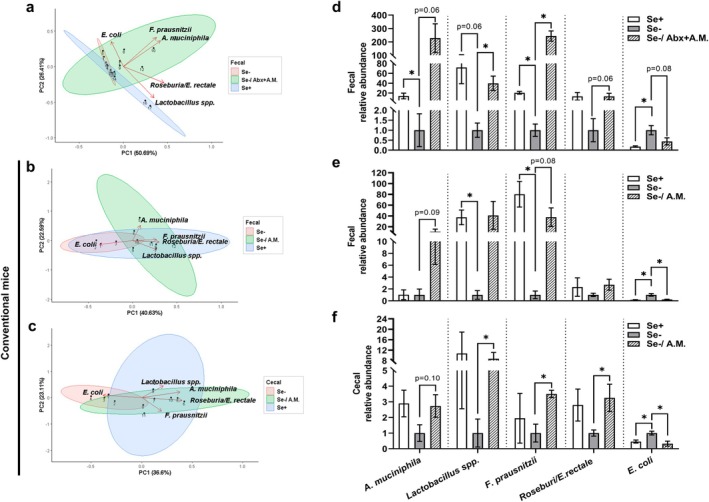
*A. muciniphila* oral gavage and dietary Se deficiency result in symbiotic and competitive changes in the relative abundance of the 5 bacterial taxa in male C57BL/6 mice. Visualization through principal component analysis (a–c) according to the 2^−ΔΔCT^ method‐quantified, qPCR‐analyzed relative abundance of the 5 bacteria in feces of antibiotic‐pretreated mice (d) and fecal and cecal samples from conventional mice (e, f). See Figures 3a and 4a for study designs. Because the ΔΔCT method invalidates the 4 groups of a 2‐factorial design to be analyzed altogether, the Se‐deficient group was used as the reference to assess the impact of Se deficiency and whether *A. muciniphila* oral gavage influences this response. Arrows for each variable (bacterium) indicate correlations with principal components, showing the direction of increasing values for each bacterium. Statistical significance for principal component analysis was assessed using permutational multivariate analysis of variance (a, Se+ vs. Se−, *p* < 0.01; Se− vs. Se−/Abx+ A.M., *p* < 0.01. b, Se+ vs. Se−, *p* = 0.10; Se− vs. Se−/A.M., *p* = 0.11. c, Se+ vs. Se−, *p* = 0.13; Se− vs. Se−/A.M., *p* = 0.06). Values (means ± SEM, *n* = 6–7) with a bracket differ (*p ≤ 0.05 or as indicated). Abx, antibiotics pretreatment; A.M., *A. muciniphila* oral gavage; Se+, Se‐adequate diet; Se−, Se‐deficient diet.

The digestion of mucin by *A. muciniphila* produces nutrients that support the growth of certain bacteria, such as butyrate‐producing *Lactobacillus* spp., *F. prausnitzii* , and *Roseburia/E. rectale* (Belzer et al. 2017; Chia et al. 2018; Dempsey and Corr 2022; Hagi and Belzer 2021; Singh et al. 2022; Zhu et al. 2021). Principal component analysis of qPCR‐determined relative abundances of these bacteria, along with the facultative *E. coli* , revealed distinct clustering in Se‐adequate and Se‐deficient groups (purple vs. red) in the feces of antibiotic‐pretreated mice (Figure 6a). Oral gavage of *A. muciniphila* (red vs. green) positively shifted the Se‐deficient cluster along PC1. A similar trend, though marginally significant, was observed in fecal and cecal samples of conventional mice (Figure 6b,c). Further analysis of the variables showed that Se‐deficient groups, with lower PC1 scores, were generally associated with *E. coli* abundance (negative values) and decoupled from the other four bacteria (positive values). In addition to this holistic view, Se deficiency reduced (*p* ≤ 0.06) relative abundances of fecal *Lactobacillus* spp. and *F. prausnitzii* in both antibiotic‐pretreated and conventional mice, but had no significant impact on their abundance in the cecum or on fecal and cecal *Roseburia/E. rectale* abundance (Figure 6d–f). In contrast, Se deficiency increased *E. coli* in all three groups, but this increase was reversed (*p* ≤ 0.08) following *A. muciniphila* gavage. *A. muciniphila* gavage also increased (*p* ≤ 0.10) the abundance of *A. muciniphila* , *Lactobacillus* spp., *F. prausnitzii* , and *Roseburia/E. rectale* in Se‐deficient mice, except for fecal *Lactobacillus* spp. and *Roseburia/E. rectale* in conventional mice. In Se‐adequate mice, *A. muciniphila* gavage modestly separated the clusters, affecting (*p* ≤ 0.07) five of 15 pairwise comparisons in relative abundance: (1) upregulation of fecal *A. muciniphila* in both groups (3 vs. 19‐fold increase), fecal *F. prausnitzii* in antibiotic‐pretreated mice, and cecal *Roseburia/E. rectale* in conventional mice; and (2) downregulation of fecal *E. coli* in conventional mice (Figure S12a–f). In *A. muciniphila* ‐administered mice, Se‐deficient and Se‐adequate clusters were marginally separated, with no clear association between specific bacteria and dietary groups. Se deficiency only increased fecal *E. coli* and decreased cecal *Roseburia/E. rectale* abundance in conventional mice (Figure S13a–f). Altogether, these results provide insights into dysbiosis following dietary Se deficiency, along with competitive suppression of *E. coli* and symbiosis with butyrate‐producing bacteria in response to *A. muciniphila* gavage in mature and middle‐aged Se‐deficient mice.

## 3 Discussion

**FIGURE 7:**
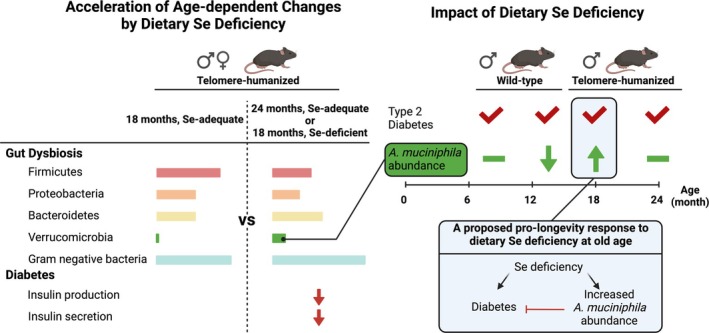
Graphic summary. All results supporting this summary are original to this article, except for the diabetes parameters in telomere‐humanized mice (Wu et al. 2017). Generation 3 *Terc* ^−/−^ telomere‐humanized mice (telomere length: 16 vs. 10–15 kb in humans) have an older biological age than wild‐type mice at the same chronological age (Samper et al. 2001).

Dietary Se deficiency and aging both induce gut dysbiosis (Kasaikina et al. 2011; Rampelli et al. 2013), but whether reshaped gut microbiota contribute to the opposing effects of Se deficiency on healthspan deterioration (e.g., type 2 diabetes) and longevity in aged mice remains unclear (Wu et al. 2017). As depicted in Figure 7, Se deficiency accelerates gut microbial changes and type 2 diabetes pathogenesis in telomere‐humanized mice aged from 18 to 24 months. *A. muciniphila* , a bacterium of the phylum Verrucomicrobia, is the most prominent species enriched with age in both sexes, but Se deficiency accelerates this enrichment only in 18‐month‐old males (Figure 2g,h). In contrast, Se deficiency does not enrich *A. muciniphila* in the gut of mature or middle‐aged male wild‐type mice, though it induces type 2 diabetes, which is alleviated by *A. muciniphila* gavage (Figures 3, 4, and 6). Since males typically age earlier than females and telomere‐humanized mice age faster than wild‐type mice (Hägg and Jylhävä 2021; Wu et al. 2017), the Se deficiency‐accelerated *A. muciniphila* enrichment likely depends on a threshold of biological age and physiological decline. Reduced mucosa thickness, which inhibits *A. muciniphila* proliferation, is observed in the colon of 7‐month‐old generation 3 *Terc* ^−/−^ telomere‐humanized mice (Qi et al. 2023), suggesting that the genetic background does not explain the *A. muciniphila* enrichment due to Se deficiency. Since *A. muciniphila* outcompetes other bacterial taxa for mucin substrates, future studies should explore how altered glycan composition affects type 2 diabetes pathogenesis in Se‐deficient older mice. We propose that Se deficiency accelerates biological aging while promoting a pro‐longevity (Yim et al. 2019) or adaptive response (Zhang et al. 2018), paradoxically resulting in *A. muciniphila* enrichment that lowers type 2 diabetes risk (Figure 7) and an unexpected reduction in the Firmicutes/Bacteroidetes ratio (Figure 1c). This may be specific to biologically very old mice, somewhat similar to the distinct genetics and metabolism of centenarians (Garagnani et al. 2013; Ying et al. 2024).

A key question is why dietary Se deficiency leads to both detrimental type 2 diabetes and beneficial *A. muciniphila* enrichment. Body Se primarily exists in selenoproteins at nutritional levels, and Se deficiency reduces their expression while retaining low molecular weight Se species, causing the latter to dominate the Se pool (Combs Jr. 2015). Since excess Se (> 10 times nutritional needs) is toxic, low doses may promote longevity through hormesis under chronic deficiency (Zhang et al. 2018). From this perspective, *A. muciniphila* enrichment is conjectured to adaptively counteract diabetes in Se‐deficient, aged mice. Supporting evidence includes: (1) a negative correlation between liver Se levels and longevity in 26 mammalian species (Ma et al. 2015); (2) mixed findings on *A. muciniphila* abundance with age in mice and humans (Biagi et al. 2016, 2010; Fransen et al. 2017; Langille et al. 2014; van der Lugt et al. 2018), suggesting biological age and/or telomere length as key factors; (3) Se deficiency increasing butyrate concentrations in males (Figure 2d), consistent with human studies negatively linking aging and SCFA contents (Woodmansey 2007).

Most reports highlight the beneficial roles of *A. muciniphila* in healthspan and lifespan, including its enrichment in centenarians from Emilia‐Romagna, Italy (Biagi et al. 2016), its ability to improve longevity in progeria mice (Bárcena et al. 2019), and its potential to reduce type 2 diabetes symptoms in obese mice (Everard et al. 2011; Shin et al. 2014) and humans (Bárcena et al. 2019; Depommier et al. 2019). Our studies show that *A. muciniphila* alleviates Se deficiency‐induced type 2 diabetes‐like symptoms in non‐obese mice at mature and middle age, suggesting that these health benefits are not limited to old age or obesity‐related diabetes. Interestingly, *A. muciniphila* gavage reshapes the gut microbiota by promoting butyrate‐producing, anti‐diabetic species (e.g., *Lactobacillus* spp., *F. prausnitzii* and *Roseburia*; Biagi et al. 2010; Karlsson et al. 2013; Xuan et al. 2023), while suppressing diabetes‐promoting facultative bacteria such as *E. coli* (Hänninen et al. 2018; Pedersen et al. 2016). Furthermore, both heat‐inactivated and live *A. muciniphila* offer similar protection against high‐fat diet‐induced type 2 diabetes in mice (Everard et al. 2013), suggesting that its metabolites may benefit the host. Future studies should explore how *A. muciniphila* alleviates diabetes in this context.

Dietary Se deficiency accelerates aging‐related gut microbial changes in aged telomere‐humanized mice in a sexually dimorphic manner. Common changes include a reduced Firmicutes/Bacteroidetes ratio, between‐group variability, and enrichment of gram‐negative bacteria like Bacteroidetes, which are elevated in diabetic patients (Crudele et al. 2023). Male‐specific changes include reduced *Clostridia*, *Actinobacteria*, and *Bifidobacterium* abundances, increased community variability (Gini–Simpson index), increased fecal butyrate levels, and *A. muciniphila* enrichment. These dimorphic effects likely result from faster male aging (Hägg and Jylhävä 2021) and sex‐specific Se metabolism and selenoprotein functions (Combs Jr. et al. 2012; Hägg and Jylhävä 2021; Méplan et al. 2007; Riese et al. 2006). For example, reduced selenoprotein‐expressing *Clostridia* and *Actinobacteria* may enhance Se bioavailability in Se‐deficient males by reducing Se competition from the gut microbiota. While β‐estradiol, but not testis‐derived hormones, enriches *A. muciniphila* , and testosterone‐producing Leydig cells are enlarged in Se‐deficient rats (Sakamuri et al. 2023; Seale et al. 2018), the impact of sex hormones on Se deficiency‐enriched *A. muciniphila* in old age remains unknown. Future studies should explore factors like metformin, which enriches *A. muciniphila* and mimics Se deficiency (Cani et al. 2022; Takayama et al. 2014), likely in a sex‐specific manner (Krysiak et al. 2016).

*A. muciniphila* comprises 3%–5% of the microbial community in healthy subjects (Cani et al. 2022). Its administration helps restore the abundance of other SCFA‐producing bacteria (*Lactobacillus*, *Roseburia*/ *E. rectale* and *F. prausnitzii* ) in Se‐deficient mice, improving gut barrier function and reducing inflammation (Figures 3, 4, 5, 6). These bacteria colonize the mucus layer, enhance barrier integrity, increase butyrate bioavailability, and reduce inflammation and *E. coli* colonization (Li et al. 2020; Panpetch et al. 2020; Qiu et al. 2013; Quévrain et al. 2016; Simeoli et al. 2015; Stecher and Hardt 2008; Van den Abbeele et al. 2013). Our findings align with studies showing an inverse correlation between plasma Se levels and gut *Enterobacteriaceae*, such as *E. coli* (Liu et al. 2022). Furthermore, butyrate production by these beneficial bacteria reduces inflammation and insulin resistance in inflammatory bowel disease patients and obese mice (Gao et al. 2009; Marchesi et al. 2007; Nemoto et al. 2012).

Lipopolysaccharide, a potent inducer of inflammation and endotoxemia, is a major component of the outer membrane of gram‐negative bacteria (Cani et al. 2008). Dietary Se deficiency increases gram‐negative bacteria in diabetic telomere‐humanized mice, but how the concurrent enrichment of *A. muciniphila* alleviates inflammation is unclear. Rather, mucin degradation by *A. muciniphila* can promote intestinal inflammation and pathogen colonization (Ganesh et al. 2013; Ng et al. 2013), and its colonization is linked to tumorigenesis in the Fabpl*Cre*;*Apc* ^15lox/+^ colorectal cancer mouse model (Dingemanse et al. 2015). Furthermore, a systematic review of randomized controlled trials found no significant effect of probiotic supplementation on gut microbiota in healthy individuals (Kristensen et al. 2016). Thus, *A. muciniphila* enrichment may benefit only certain diseased states, such as diabetes, plausibly by facilitating metabolite flux into the host for glucose homeostasis.

Antibiotic treatment has been shown to improve type 2 diabetes symptoms in leptin‐deficient (*ob*/*ob*) and diet‐induced obese mice (Chou et al. 2008), possibly by reducing SCFA levels, which prompts intestinal cells to use glucose to compensate for energy deficits (Zarrinpar et al. 2018). While awaiting verification under gnotobiotic conditions, our findings extend these results to non‐obese diabetic mice and show that *A. muciniphila* is more effective in alleviating type 2 diabetes‐like symptoms in antibiotic‐pretreated than in conventional mice, supporting the notion of dysbiosis with pro‐diabetes gut microbiota due to Se deficiency. However, antibiotic treatment may also promote pathogen proliferation and virulence under normal conditions in humans (Perez‐Lopez et al. 2016; Schubert et al. 2015).

Se‐utilizing and selenoprotein‐expressing bacteria, such as *E. coli* , *Clostridia*, and *Enterobacteria*, can colonize the gastrointestinal tract (Hrdina et al. 2009; Kasaikina et al. 2011), competing with the host for dietary Se, which reduces Se bioavailability and selenoprotein expression in the host. However, the four *Akkermicia* species we tested lack selenoprotein and Se‐utilization genes, suggesting that this probiotic does not jeopardize dietary Se, especially when intake is low. Future studies should explore how *A. muciniphila* alleviates type 2 diabetes, focusing on microbiota‐modulated metabolites, inflammasome signaling (Levy et al. 2015; Nowarski et al. 2015), and Se status in both host and microbiota. These studies should also extend to other type 2 diabetes models (e.g., high‐fat diet or defective leptin homeostasis) and aging to improve translational relevance.

In conclusion, optimizing gut microbiota through adequate dietary Se may help prevent or manage early aging and type 2 diabetes. Se deficiency accelerates age‐related gut microbiota changes in diabetic telomere‐humanized mice at old age, with *A. muciniphila* being most enriched. Given the proposed pro‐longevity effects of Se deficiency (Wu et al. 2017; Yim et al. 2019), the increased presence of *A. muciniphila* could adaptively mitigate type 2 diabetes, but further research is needed on its protective mechanisms via bacterial metabolites, especially SCFAs and selenoproteins. Nonetheless, caution is advised when supplementing *A. muciniphila* in healthy individuals, as it may inadvertently cause hypoglycemia.

## 4 Methods

### 4.1 Mouse Studies

All mice used in the three studies were on the C57BL/6J background and were fed torula yeast‐based purified diets. For bacterial *16S rRNA* gene amplicon analysis (see Figure 1a for study design), generation 3 *Terc* ^*−/−*^ telomere‐humanized mice—bred from *Terc* ^+/−^ mice (#004132, the Jackson Laboratory, Bar Harbor, ME)—were fed as previously described, and the fecal samples were collected from the same study (Wu et al. 2017). For the *A. muciniphila* oral gavage studies (see Figures 3a and 4a for study design), wild‐type mice were fed the same diets (Huang et al. 2021), except that the diets were irradiated.

In the antibiotic pretreatment studies (Figure 3a), middle‐aged male mice (11 months) were fed Se‐deficient or Se‐adequate diets for 5 weeks, followed by oral gavage with either a sham (sterile PBS) or an antibiotic solution (200 μL containing vancomycin, neomycin, and metronidazole at 50, 100, and 100 mg/kg body weight, respectively) every 12 h, and ampicillin (1 mg/mL) *ad libitum* in drinking water for 1 week (Reikvam et al. 2011). Mice then received two oral gavages, either sham or 1 × 10^9^ CFU live *A. muciniphila* in 200 μL anaerobic PBS with 2.5% glycerol, at weeks 6 and 7. This study aimed to preliminarily assess the impact of *A. muciniphila* enrichment on Se deficiency‐induced type 2 diabetes symptoms. To reduce confounding effects from preexisting bacteria, mice assigned to receive *A. muciniphila* were pretreated with antibiotics; however, comparisons between *A. muciniphila* and sham under antibiotic conditions (i.e., Se−/Abx vs. Se−/Abx + A.M.) were not performed due to antibiotic‐induced increases in selenoprotein expression (Hrdina et al. 2009; Kasaikina et al. 2011).

In the conventional male mice cohort (Figure 4a), 2‐month‐old mice were fed Se‐deficient or Se‐adequate diets for 21 weeks, followed by daily oral gavage with sham or 2 × 10^8^ CFU live *A. muciniphila* in 200 μL for 4 weeks (Everard et al. 2013), aiming to reshape the gut microbiota through *A. muciniphila* supplementation.

Altogether, the latter two studies aimed to investigate the causal relationship between *A. muciniphila* and the protection against Se deficiency‐induced type 2 diabetes‐like symptoms. While not directly addressing it, they also examined how the role of *A. muciniphila* might vary based on environmental factors (antibiotic pretreatment vs. none) and age (8 vs. 13 months). The *A. muciniphila* ‐administered mice were handled aseptically and housed individually in sterile, ventilated cages in a specific pathogen‐free room (12‐h light/dark cycle). Water and wood‐shaving bedding were sterilized by autoclaving. Body weight and food intake were recorded weekly. Two days after completing insulin sensitivity assays, mice were fasted for 6 h (from 8 a.m. to 2 p.m.), anesthetized with carbon dioxide, and euthanized by exsanguination via cardiac puncture. Liver, skeletal muscle, intestinal segments, and fresh fecal and cecal contents were collected, immediately frozen in liquid nitrogen, and stored at −80°C for further analysis. All procedures were approved by the Institutional Animal Care and Use Committees of the University of Maryland at College Park and Mississippi State University.

### 4.2 *A. muciniphila* Multiplication

*A. muciniphila* MucT (ATCC BAA‐835) was cultured anaerobically using the Anoxomat III Jar system (Advanced Instruments, Norwood, MA, USA) in a mucin‐based brain heart infusion medium (Derrien et al. 2004). The cultures were washed and prepared anaerobically in sterile anaerobic PBS with 25% (vol/vol) glycerol to final concentrations of 5 × 10^10^ (antibiotic‐treated mice) or 1 × 10^10^ (conventional mice and pigs) CFU/mL (Everard et al. 2013), then immediately frozen and stored at −80°C. Prior to oral gavage, the glycerol stocks were thawed and diluted to the required concentrations.

### 4.3 Bacterial Genomic DNA Extraction and Sequencing

DNA was extracted from fecal samples using the QIAamp PowerFecal Pro DNA Kit (#51804, QIAGEN, Germantown, MD, USA) and amplified with universal primers (Table S1) targeting the V3‐4 regions of the bacterial 16S rRNA gene. PCR products were quantified using a spectrophotometer (ND‐2000; NanoDrop Technologies, Wilmington, DE, USA) and assessed for quality via 1% agarose gel electrophoresis. Free and dimerized primers were removed using AMPure XP beads (Beckman Coulter, Brea, CA, USA). 16S rRNA sequencing libraries were generated using the NexteraXT Index Kit (Illumina, San Diego, CA, USA), with index codes added according to the manufacturer's instructions. Library quality was analyzed using the Qubit fluorometer (Invitrogen, Carlsbad, CA, USA) and the Agilent Bioanalyzer 2100 system (Agilent, Palo Alto, CA, USA), with the final library trace around 630 bp. The libraries were normalized to 30 nM using Qubit readings, pooled, and diluted to 10 nM for storage. They were then denatured with NaOH, diluted with hybridization buffer, and sequenced with paired‐end 300 bp reads on the Illumina MiSeq system (Illumina, San Diego, CA, USA) at Mississippi State University's Institute for Genomics, Biocomputing, and Biotechnology.

### 4.4 Analysis of 16S rDNA Sequencing Data

The raw mate‐paired FASTQ files were quality‐filtered and analyzed using Illumina MiSeq Reporter Software v2.3. Reads were classified against the Greengenes database mainly at the genus level, with 15%–20% of bacteria recognized at the species level. A total of 34,509,237 high‐quality sequencing reads were obtained from 48 samples (*n* = 6). Principal component analysis was conducted using classical multidimensional scaling on a Pearson covariance distance matrix, based on per‐sample normalized classification abundance vectors. The results showed similarity in relative abundance across samples. Heatmaps of the top 25 genera were generated using the pheatmap algorithm and Canberra distance metric in R (version 4.4). Cluster decoupling was statistically tested using Fisher's Exact test. The Shannon and Gini‐Simpson indices were calculated primarily at the genus level.

### 4.5 cDNA Synthesis and Bacterial DNA Isolation for qPCR Analysis

Total RNA from tissues was isolated in the presence of DNase I using Quick‐RNA Miniprep Kit (#R1058, Zymo Research, Irvine, CA, USA) according to the instructions. One μg of RNA was treated with dsDNase and used for first‐strand cDNA synthesis with oligo(dT)_18_ and random hexamer primers (Maxima H Minus cDNA Synthesis Master Mix, #M1681, Thermo Scientific, Waltham, MA, USA), followed by storage at −80°C. Bacterial DNA was isolated from fecal and cecal samples using the QIAGEN #51804 kit. *Rpl19* was used as the internal control for RT‐qPCR analysis of intestinal samples, while universal 16S rDNA primer was used as a proxy to quantify total bacteria. Primer sequences are detailed in Extended Data Table S1. Using PowerUp SYBR Green Master Mix (#A25741, Applied Biosystems, Waltham, MA, USA), DNAs were amplified under the following conditions: 95°C for 2 min, then 40 cycles of 95°C for 5 s and 60°C for 30 s on a QuantStudio 3 or 5 Real‐Time PCR System (Applied Biosystems). For absolute quantification (Figures 2h and S1), standard curves were created from 10‐fold serial dilutions of the PCR product‐ligated vectors to calculate the concentration of each sample by comparing the crossing point values. Other samples were analyzed using the 2^−ΔΔCT^ relative abundance method (Livak and Schmittgen 2001). Because the second ΔCT normalized the first ΔCT values by subtracting the control group, two‐way ANOVA was invalid for analyzing 2^−ΔΔCT^ relative abundance values, as a designated control group could not be applied to the group that differed by two factors. Thus, the 2^−ΔΔCT^ values were analyzed by unpaired *t*‐test and used for principal component analysis (Figures 6a–c and S12–S13).

### 4.6 Immunoblotting

Tissues were homogenized in RIPA lysis solution containing protease inhibitors (#sc‐24,948, Santa Cruz Biotech, Dallas, TX, USA), and then centrifuged at 12,000× *g* for 10 min at 4°C. Total protein concentration was determined using the Pierce BCA Protein Assay Kit (#23225, Thermo Scientific). Supernatants (30 μg protein per lane) were loaded onto and separated by 14% or 10% SDS‐PAGE (for AKT, p‐AKT S473, and p‐AKT T308). Proteins were transferred to polyvinylidene difluoride membranes and incubated overnight at 4°C with primary antibodies (Table S2), followed by incubation with HRP‐conjugated secondary antibodies for 2 h at room temperature. Images were developed using Clarity Western ECL substrate, captured with the ChemiDoc‐XS system, quantified using the volume tool in Image Lab Software (Bio‐Rad, Hercules, CA, USA), and normalized to albumin, β‐tubulin, or AKT. All original blots are shown either in main figures or extended data (Figures 3, 4, S4, S6, S7, and S9).

### 4.7 Immunohistochemistry

Formalin‐fixed, paraffin‐embedded blocks were sectioned at 4 μm onto positively charged slides, deparaffinized, and rehydrated. Slides were retrieved with proteinase K (5 min), followed by an endogenous peroxidase block (3% hydrogen peroxide, 10 min) and a background block (Background Buster, Innovex Biosciences, Richmond, CA, USA). Primary antibodies were applied for 1 h at room temperature. After rinsing, slides were incubated with DAKO Envision HRP System reagent (30 min), developed with DAKO DAB Plus (5 min), followed by DAB Enhancer (3 min), rinsed in distilled water, counterstained, and cover‐slipped. A negative control slide was processed similarly, omitting the primary antibody.

### 4.8 Glucose Tolerance and Insulin Sensitivity

Mice were fasted for 8 h before receiving an intraperitoneal injection of glucose (1 g/kg body weight) or insulin (0.25 unit/kg body weight; both Sigma–Aldrich). A drop of blood from the tail vein was collected to measure glucose concentrations before and 0.25–2 h after the injections. Insulin sensitivity assays were performed 2 days after the glucose tolerance tests. Blood glucose levels were determined using a glucose meter (Bayer Contour Next EZ, Ascensia Diabetes Care US Inc., Parsippany, NJ, USA).

### 4.9 Serum Markers and Short‐Chain Fatty Acids

Serum triglycerides, glucose, total cholesterol, LDL, and HDL were quantitated using a Cobas Integra 400 Plus analyzer (Roche Diagnostics, Basel, Switzerland). Serum insulin and lipopolysaccharide levels were quantified using ELISA kits (80‐INSMS‐E0, ALPCO Diagnostics, Salem, NH; MBS452438, MyBioSource, San Diego, CA, USA). Fecal SCFA concentrations were determined as described previously by gas chromatography with flame‐ionization detection (Thermo Trace‐1310 equipped with a TriPlus RSH Autosampler) using a DB‐FFAP 125–3237 fatty acid phase column (Agilent Technologies) (Zeng et al. 2018).

### 4.10 Mucus Layer Thickness

Following exsanguination, proximal colon segments were collected, immediately fixed in Carnoy's solution (#C2720, LabAlley, Austin, TX, USA) for 2 h at 4°C, and then immersed in 100% ethanol for 24 h. Paraffin sections (5 μm) were stained with Alcian blue. A minimum of 20 measurements were made perpendicular to the inner mucus layer per field. Twenty to forty randomly selected fields were analyzed per colon, totaling 1143 measurements, using ImageJ software.

### 4.11 Genomic Searches for Selenoproteins and Selenium Utilization Markers

The reference genome assemblies for four *Akkermansia* species were downloaded from NCBI GenBank: *A. muciniphila* , *A. biwaensis* , *A. glycaniphila*, and *A. massiliensis* (GCA_009731575.1, GCF_026072915.1, GCF_900097105.1, GCF_018847095.1). The genomes were searched for orthologs of known bacterial selenoprotein families (Zhang et al. 2022) using the Selenoprofiles software (version v4.5.2, selenoprofiles data v1.1.1), available at https://github.com/marco‐mariotti/selenoprofiles (Mariotti and Guigó 2010; Santesmasses et al. 2018; Ticó et al. 2024). The same software was used to search for profile alignments of Se utilization markers (Manta et al. 2022): *selD* (selenophosphate synthetase), required for all four known forms of Se utilization; *ybbB* (tRNA 2‐selenouridine synthase), required for the usage of Se as a tRNA modification; and *yqeB* and *yqeC*, putatively required for the usage of a Se‐containing cofactor in molybdenum hydroxylases. Lastly, the software Secmarker v0.4a (Santesmasses et al. 2017) was used to search for *tRNASec*, required for Sec biosynthesis.

### 4.12 Statistical Analyses

Data were analyzed using two‐way ANOVA with Tukey's post hoc tests, except for ΔΔCT‐based qPCR analyses, which were performed with an unpaired *t*‐test using SAS (version 9.4), GraphPad Prism (version 8.0), and/or XLSTAT. Heatmap, principal component analysis, Fisher's Exact test, and permutational multivariate analysis of variance were conducted using the R statistical package (version 4.4). The level of significance (*α*) was set at 0.05 (*p* ≤ 0.05), unless indicated otherwise. Final values are presented as means ± SEM.

## Author Contributions

Conceptualization: W.‐H.C. and G.F.C.J. Methodology: Y.‐C.H., C.‐Y.H., H.Z., R.N., and W.‐H.C. Investigation: Y.‐C.H., H.‐Y.L., L.Z., A.O., C.L., H.Z., R.T.Y.W., S.C., Q.W., M.T., and M.M. Visualization: Y.‐C.H., H.‐Y.L., L.Z., A.O., C.‐Y.H., T.‐L.W., and X.Z. Supervision: W.‐H.C. Writing – original draft: Y.‐C.H. and H.‐Y.L. Writing – review and editing: W.‐H.C. Funding acquisition: H.Z. and W.‐H.C. All authors contributed to the article and approved the submitted version.

## Disclosure

All the animal studies were approved by the universities and in accordance with institutional guidelines.

## Conflicts of Interest

The authors declare no conflicts of interest.

## Supporting information


Appendix S1.

Appendix S2.

## Data Availability

The raw Miseq reads generated in this study have been deposited in the Sequence Read Archive database under accession number PRJNA1083232.
