# Supplementary material for: Dietary Selenium Deficiency Accelerates the Onset of Aging‐Related Gut Microbial Changes in Aged Telomere‐Humanized Mice, With Akkermansia muciniphila Being the Most Prominent and Alleviating Selenium Deficiency‐Induced Type 2 Diabetes
Source: Aging Cell. 2025 Jun 20;24(8):e70130. doi: 10.1111/acel.70130 (PMC12341817; doi:10.1111/acel.70130)
Supplement: Supplementary file 1 — Appendix S1. [file ACEL-24-e70130-s002.pdf]

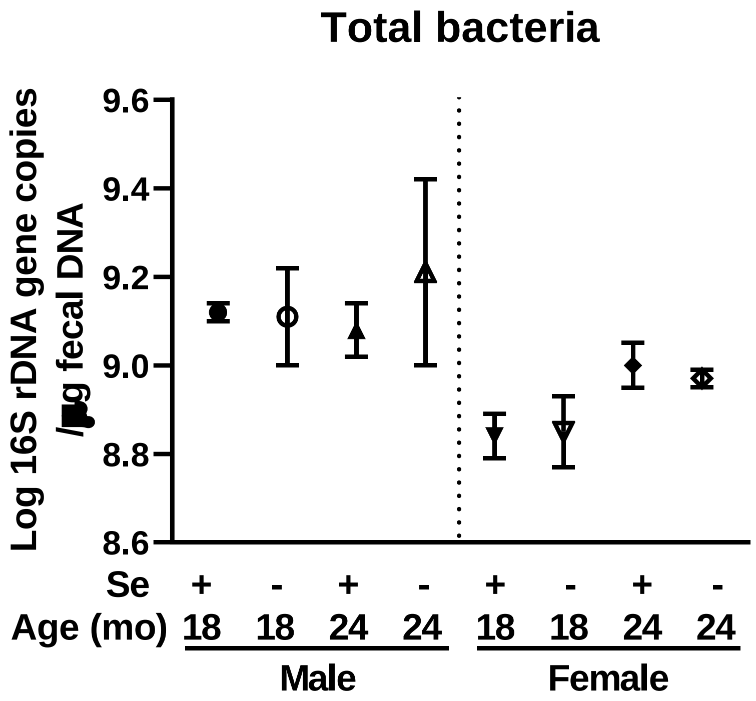

Figure S1. Total bacteria abundance

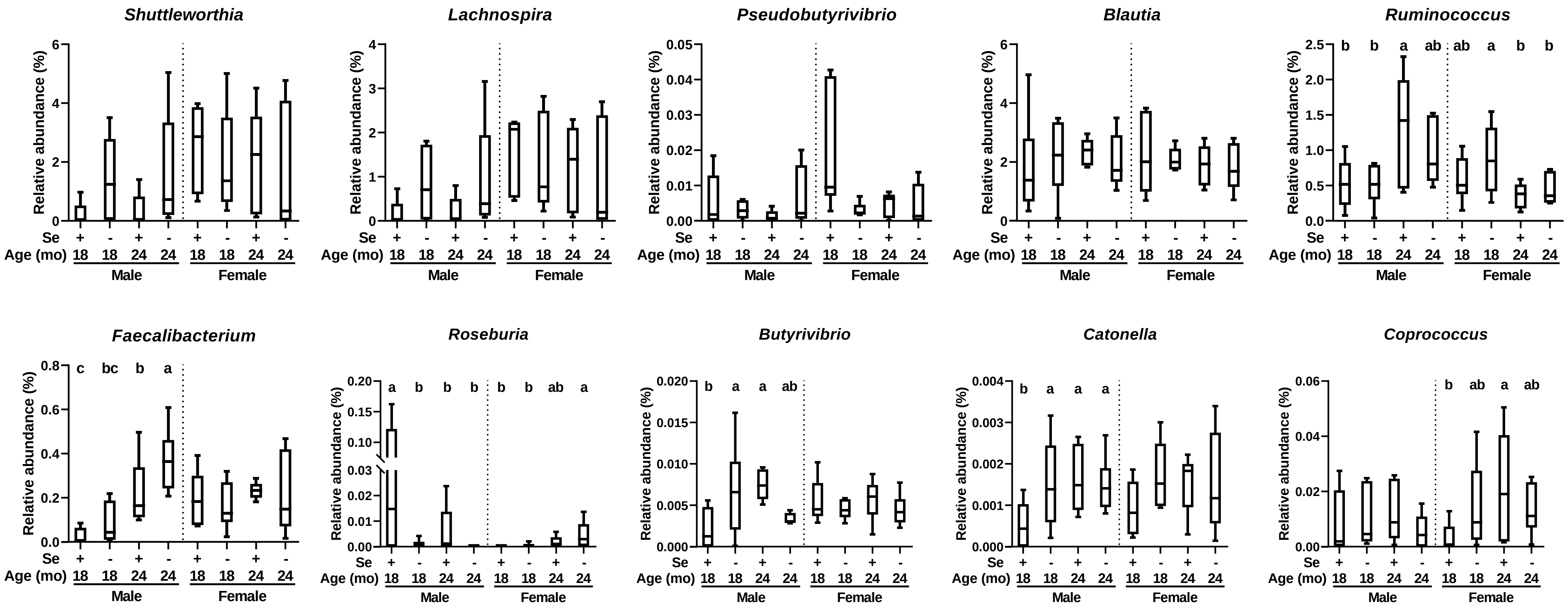

Figure S2. Relative abundance of the 10 *Clostridium* clusters IV and XIVa bacteria in fecal samples collected from Se-deficient and Se-adequate male and female telomere-humanized mice aged 18 and 24 months. Values (means ± SEM, n = 6) without sharing a common letter within a sex differ, $P < 0.05$. Se+, Se-adequate diet; Se-, Se-deficient diet.

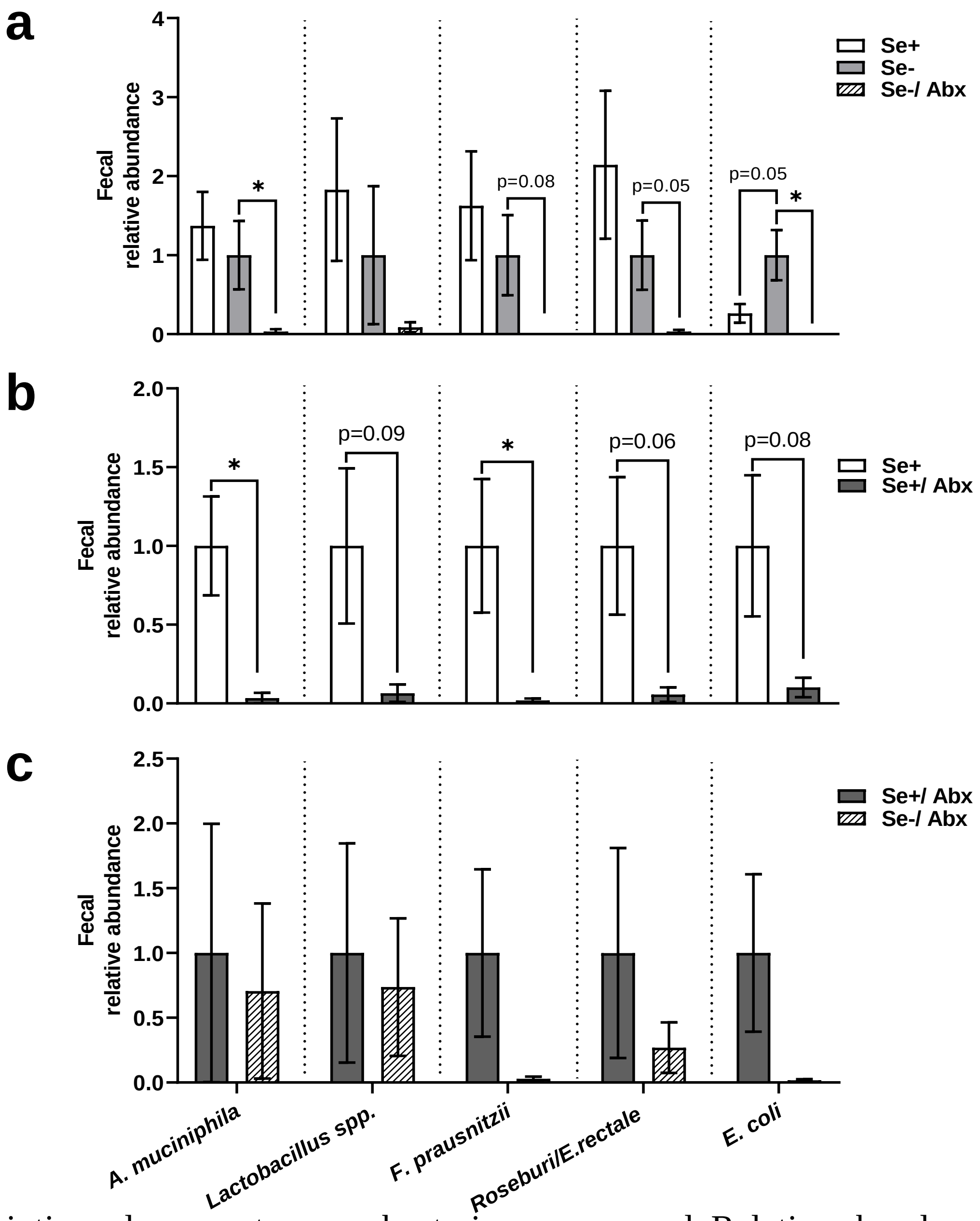

Figure S3. The efficacy of antibiotic oral gavage to purge bacteria was assessed. Relative abundances of the bacteria were determined by qPCR and quantified by the $2^{-\Delta\Delta CT}$ method, with fecal samples from mice in the designated treatment groups (see Figure 3a for study design and Table S1 for primers). *, $P < 0.05$. Abx, antibiotics pre-treatment; Se+, Se-adequate diet; Se-, Se-deficient diet.

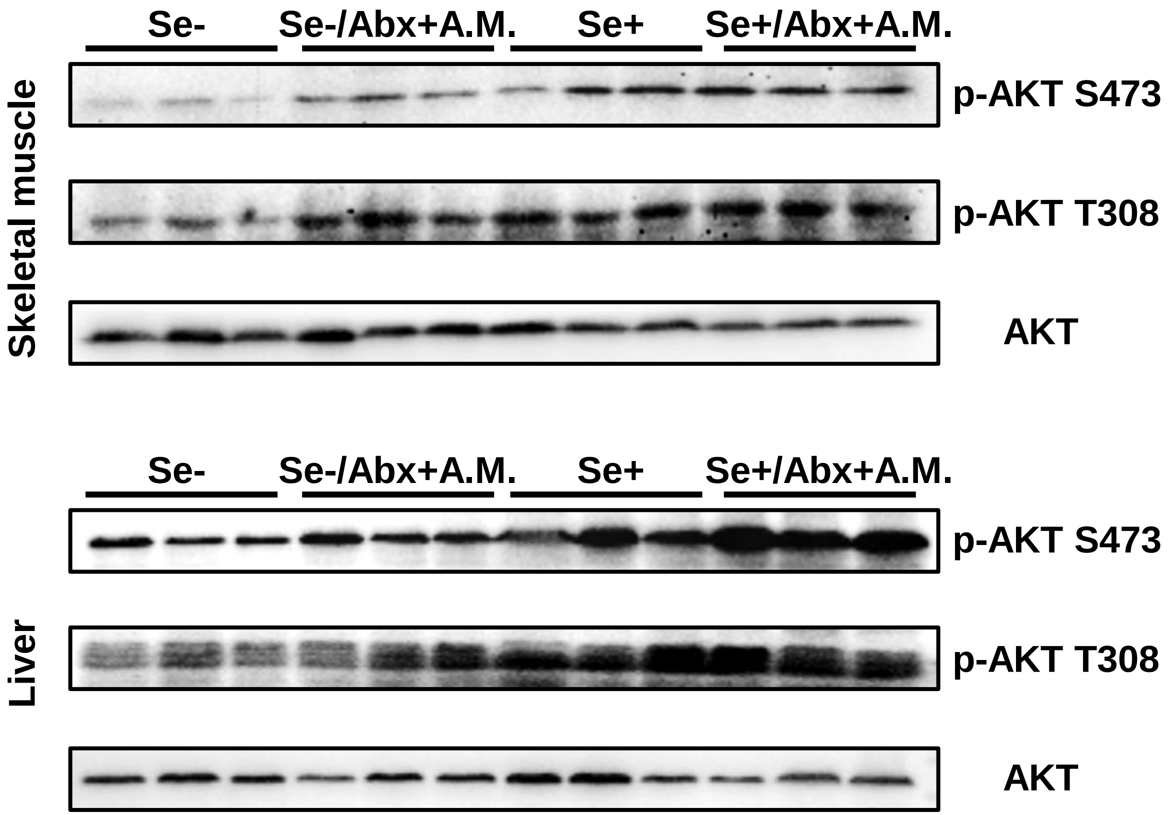

Figure S4. These are the second set of original blots (3 mice per group per blot) used for quantitative analysis, along with the other set shown in Figure 3f,g. Abx, antibiotic pre-treatment; AKT, mouse thymoma viral protooncogene; A.M., *A. muciniphila;* Se+, Se-adequate diet; Se-, Se-deficient diet.

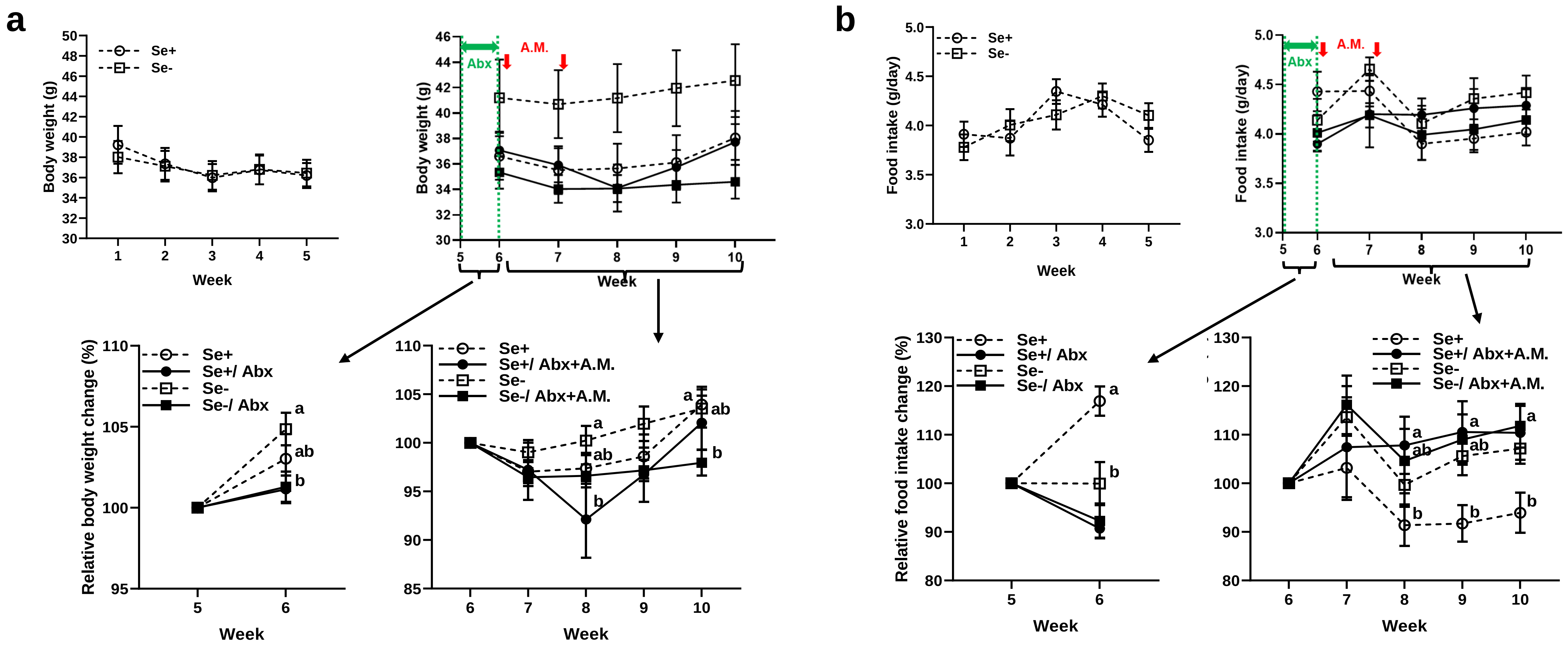

Figure S5. Body weight (**a**) and food intake (**b**) in 11-month-old mice fed a Se-deficient or Se-adequate diet, treated with antibiotics at week 5, and orally administered live *A. muciniphila* (1×10^9 CFU) at weeks 6 and 7 (see Figure 3a for detailed experimental design). Mice were individually assigned at week 0 to receive either an *A. muciniphila* or a mock oral gavage; however, body weight and food intake were variable in the four treatment groups at the time of antibiotic treatment (week 5) and *A. muciniphila* oral gavage (week 6). Therefore, relative changes were used for statistical analyses. Values (means ± SEM, n = 6) that do not share a common letter differ, $P \leq 0.05$. Abx, antibiotic treatment; A.M., *A. muciniphila*; Se+, Se-adequate diet; Se-, Se-deficient diet.

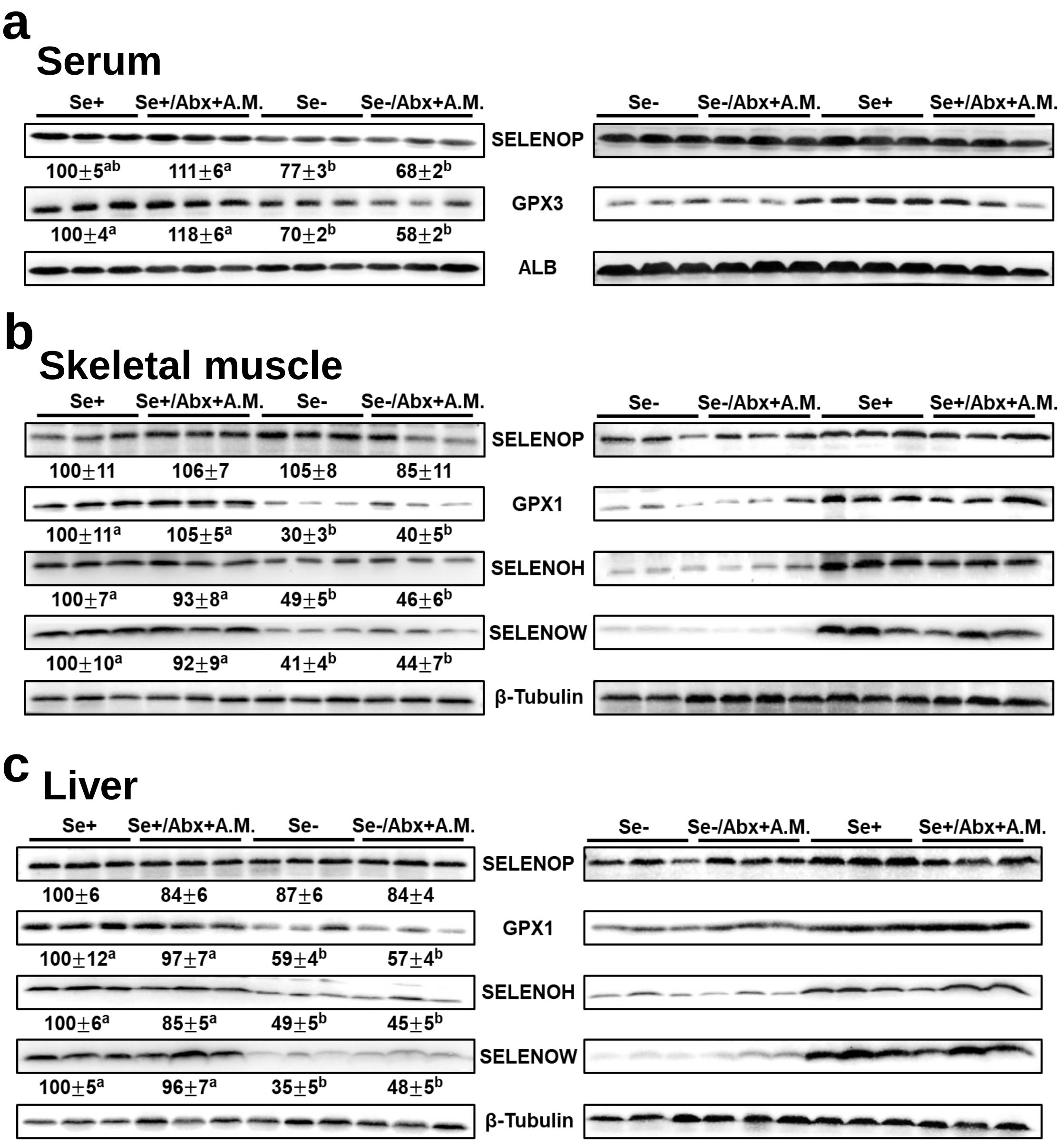

Figure S6. Western analyses of body Se status in antibiotic-treated male Se-deficient and Se-adequate C57BL/6 mice, with or without *A. muciniphila* oral gavage (1×10^9 CFU), in the serum (**a**), skeletal muscle (**b**), and liver (**c**). Band intensities in both the left and right panels were normalized to those of ALB or β-tubulin within the same blot, expressed as a percentage of the Se+ control group, and are shown in the left panels. Values (means ± SEM, n = 6) that do not share a common letter differ, $P \leq 0.05$. Abx, antibiotic treatment; ALB, albumin; A.M., *A. muciniphila*; GPX1, glutathione peroxidase 1; GPX3, glutathione peroxidase 3; Se+, selenium-adequate diet; Se−, selenium-deficient diet; SELENOH, selenoprotein H; SELENOP, selenoprotein P; SELENOW, selenoprotein W.

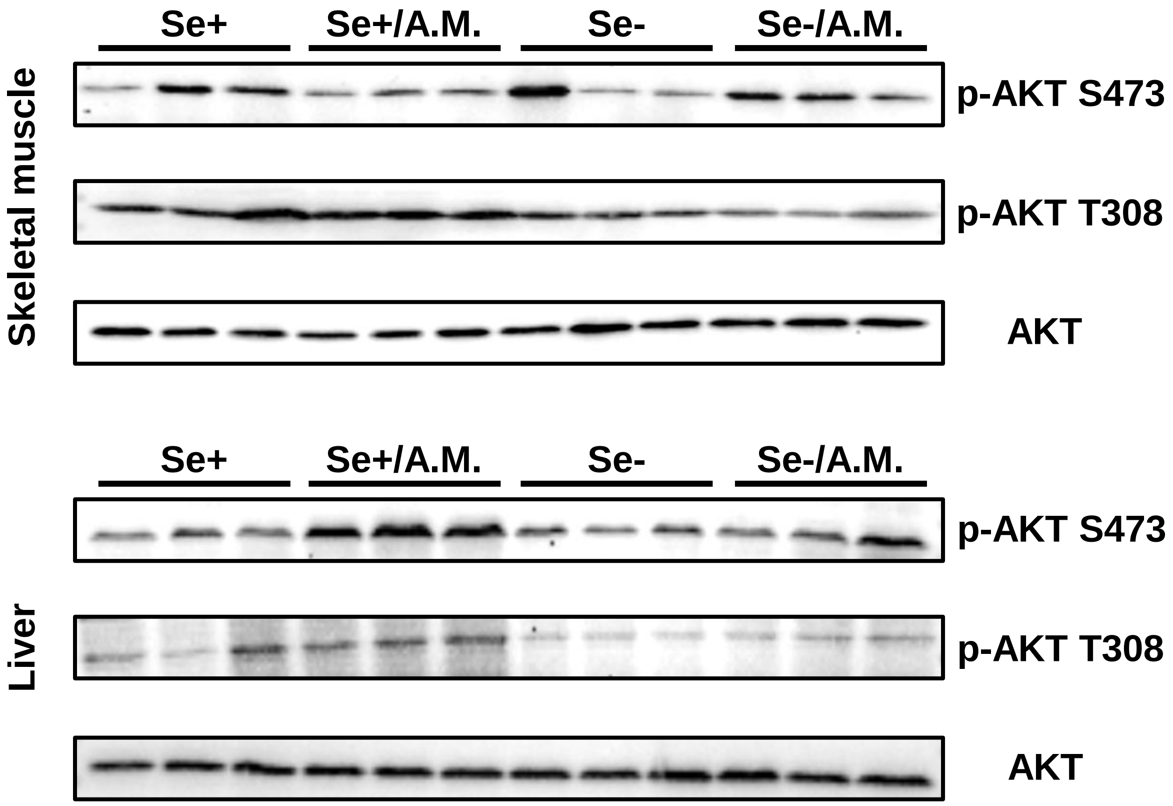

Figure S7. These are the second set of original blots (3 mice per group per blot) used for quantitative analysis, along with the other set shown in Figure 4f,g. AKT, mouse thymoma viral protooncogene; A.M., *A. muciniphila;* Se+, Se-adequate diet; Se-, Se-deficient diet.

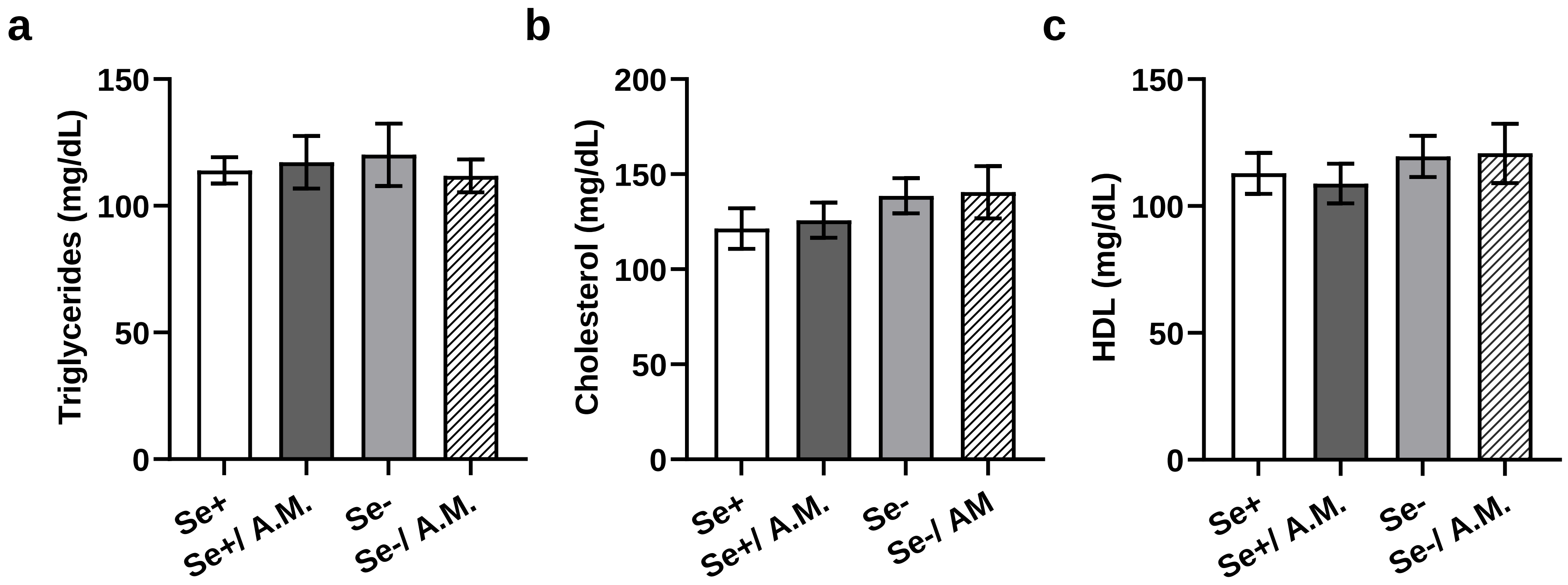

Figure S8. Fasting postmortem serum triglycerides (**a**), cholesterol (**b**), and HDL (**c**) concentrations in 8-month-old conventional male C57BL/6J mice fed either a Se-deficient or a Se-adequate diet and given *A. muciniphila* (2×10^8 CFU) or a mock oral gavage (see Figure 4A for detailed design). Values (means ± SEMs, n = 6-7) without sharing a common letter differ, $P \leq 0.05$. A.M., *A. muciniphila*; HDL, high-density lipoprotein; Se+, selenium-adequate diet; Se−, selenium-deficient diet.

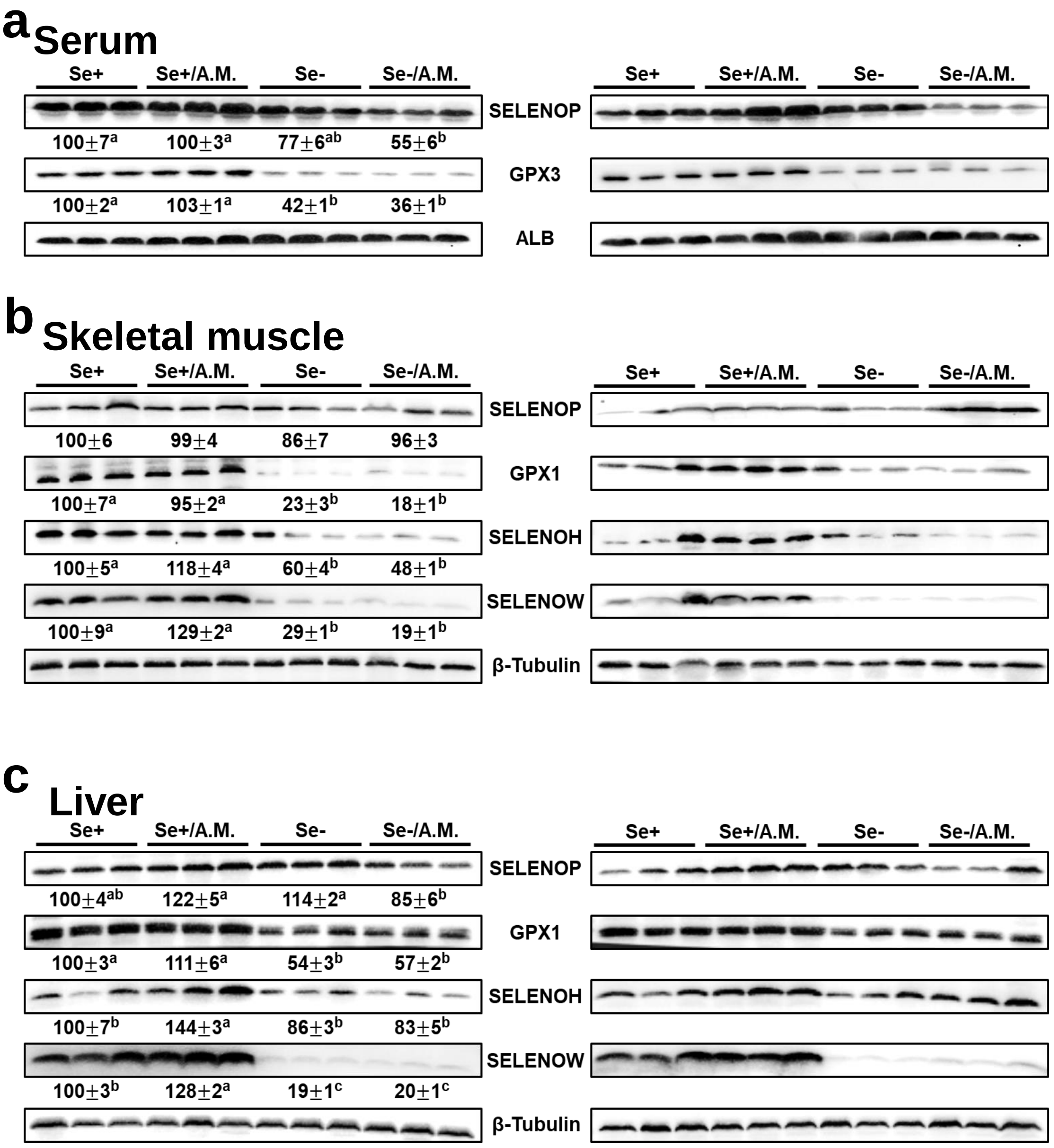

Figure S9. Western analyses of body selenium status in the serum (**a**), skeletal muscle (**b**), and liver (**c**) in conventional male C57BL/6 mice fed either a Se-deficient and a Se-adequate diet and given *A. muciniphila* (2×10^8 CFU) or mock oral gavage (see Figure 4A for detailed design). Band intensities in both the left and right panels were normalized to those of ALB or β-tubulin within the same blot, expressed as a percentage of the Se+ control group, and are shown in the left panels. Values (means ± SEMs, n = 6) without sharing a common letter differ, $P \leq 0.05$. ALB, albumin; A.M., *A. muciniphila*; GPX1, glutathione peroxidase 1; GPX3, glutathione peroxidase 3; Se+, selenium-adequate diet; Se−, selenium-deficient diet; SELENOH, selenoprotein H; SELENOP, selenoprotein P; SELENOW, selenoprotein W.

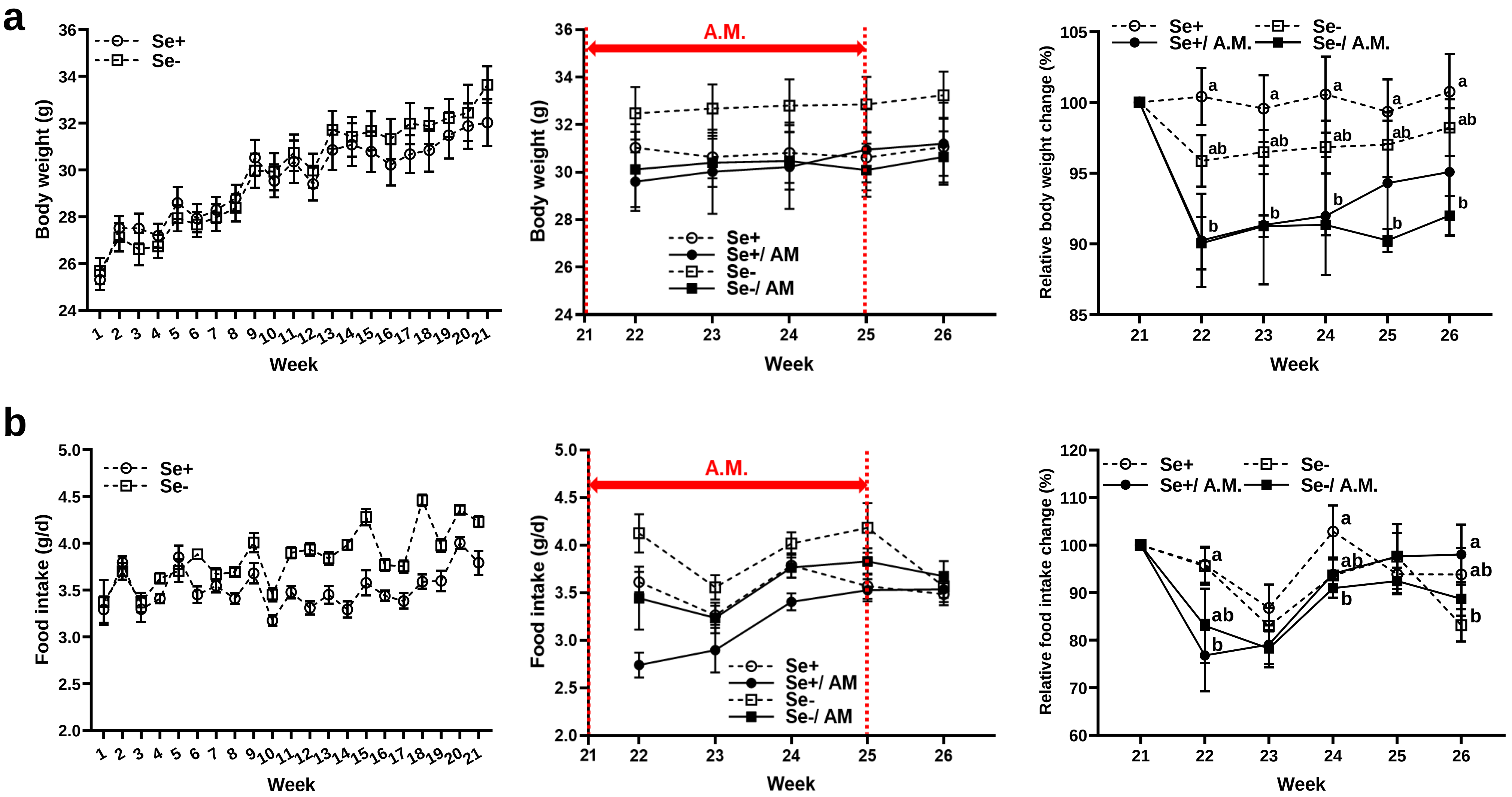

Figure S10. Body weight (**a**) and food intake (**b**) in conventional male C57BL/6 mice fed a selenium-deficient or a selenium-adequate mice at 2 months of age and orally administrated with live *A. muciniphila* (2×10^8 CFU) daily between 21 and 25 weeks after the dietary selenium manipulation (See Figure 4A for detailed experimental design). Mice were individually assigned at week 0 to receive an *A. muciniphila* or a mock oral gavage; however, body weight and food intake were variable in the 4 treatment groups at the time of *A. muciniphila* oral gavage (week 21).Therefore, relative changes in weeks 21-26 were used for statistical analyses. Values (means ± SEM, n = 6-7) without sharing a common letter differ, $P \leq 0.05$. A.M., *A. muciniphila*; Se+, Se-adequate diet; Se-, Se-deficient diet.

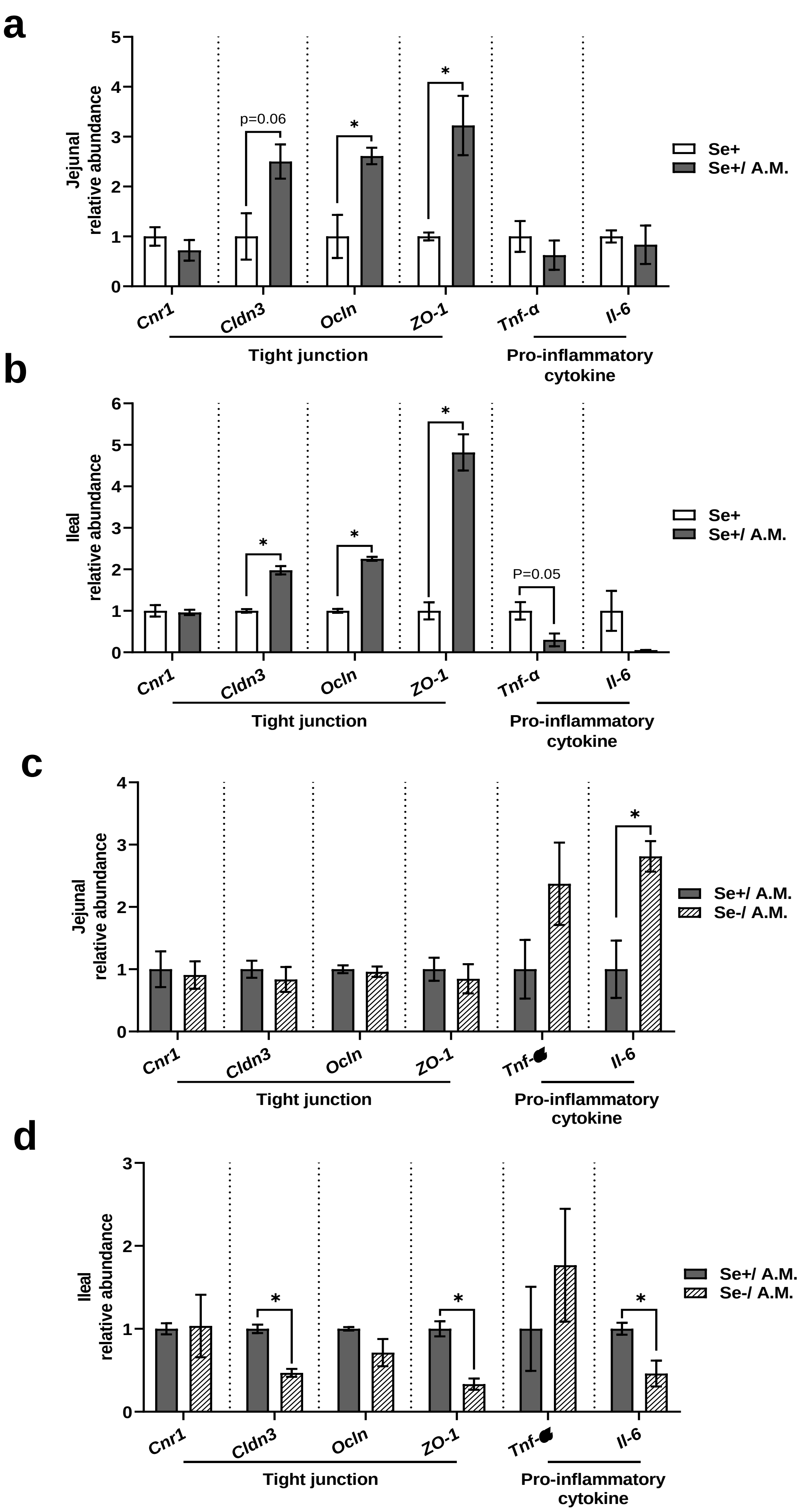

Figure S11. Tight junction and pro-inflammatory cytokine mRNA expression in the jejunum and ileum according to RT-qPCR analyses. The results are shown as pairwise comparisons: Se+ vs. Se+/A.M. (**a**,**b**) and Se+/A.M. vs. Se-/A.M. (**c**,**d**). The other two pairwise comparisons (Se- vs. Se+; Se- vs. Se-/A.M.) are shown in Fig. 5**d**,**f**. Values (means ± SEM, n = 6-7) with a bracket differ (*$P \leq 0.05$ or indicated otherwise). A.M., *A. muciniphila*; *Cnr1*, cannabinoid receptor 1; *Cldn3*, claudin 3; *Il-6*, interleukin 6; *Ocln*, occludin; Se+, selenium-adequate diet; Se−, selenium-deficient diet; *Tnf-α*, tumour necrosis factor α; *ZO*-1, zonula occludens-1.

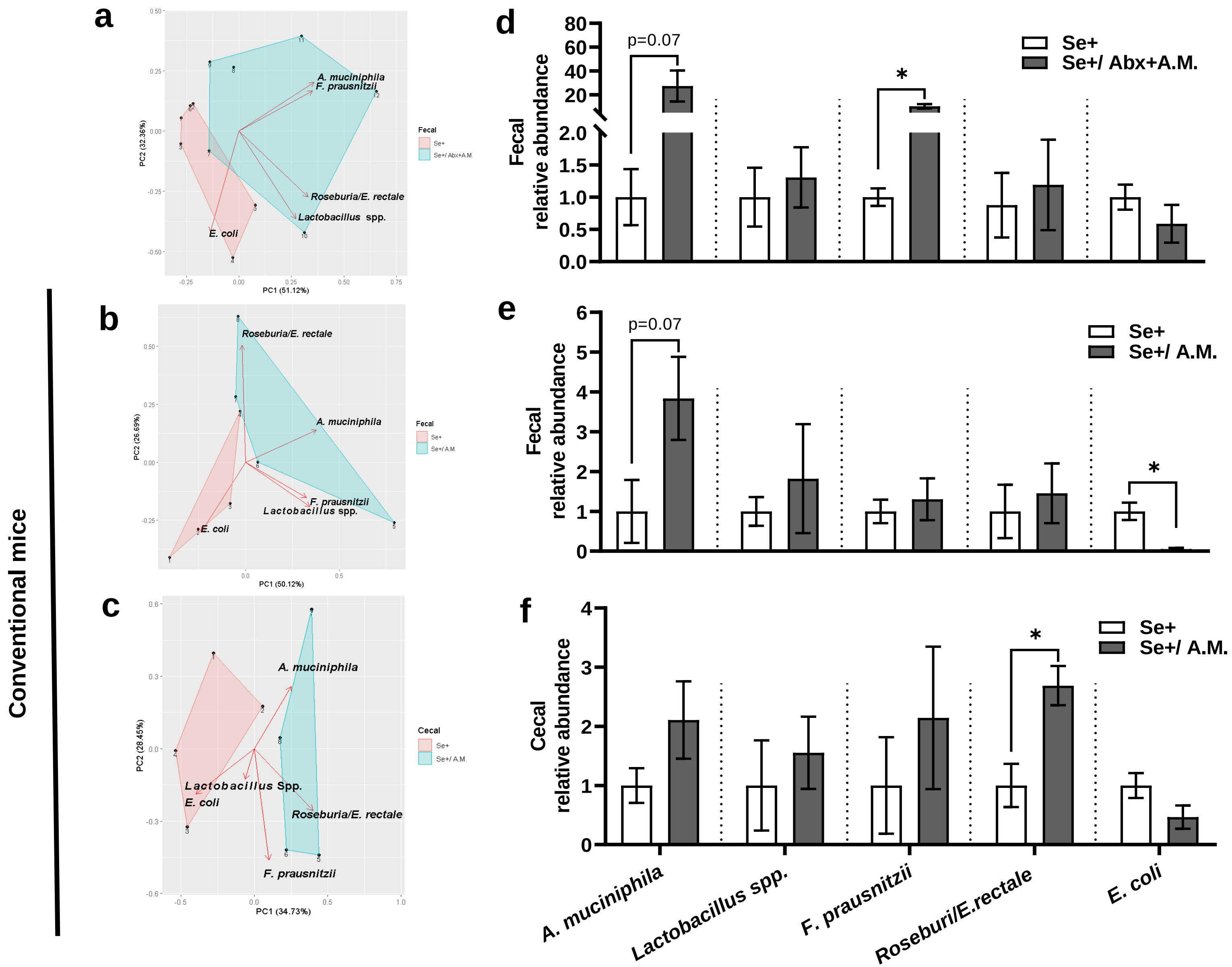

Figure S12. Visualization through principal component analysis (**a-c**) according to the $2^{-\Delta\Delta CT}$ method-quantified, qPCR-analyzed relative abundance of the 5 bacteria in feces of antibiotics-treated mice (**d**) and fecal and cecal droppings of conventional mice (**e**,**f**). See Fig. 3**a** and 4**a** for study designs. The results are shown as pairwise comparisons of Se+ vs. Se+/Abx+A.M. or Se+/A.M. The most critical pairwise comparisons (Se- vs. Se+; Se- vs. Se-/A.M.) are shown in Fig. 6. The arrows for each variable (bacterium) indicate the correlations with the principal components and show the direction of increasing values for each bacterium. Values (means ± SEM, n = 6-7) with a bracket differ (*$P \leq 0.05$ or indicated otherwise). Abx, antibiotics pre-treatment; A.M., *A. muciniphila* oral gavage; Se+, Se-adequate diet; Se-, Se-deficient diet.

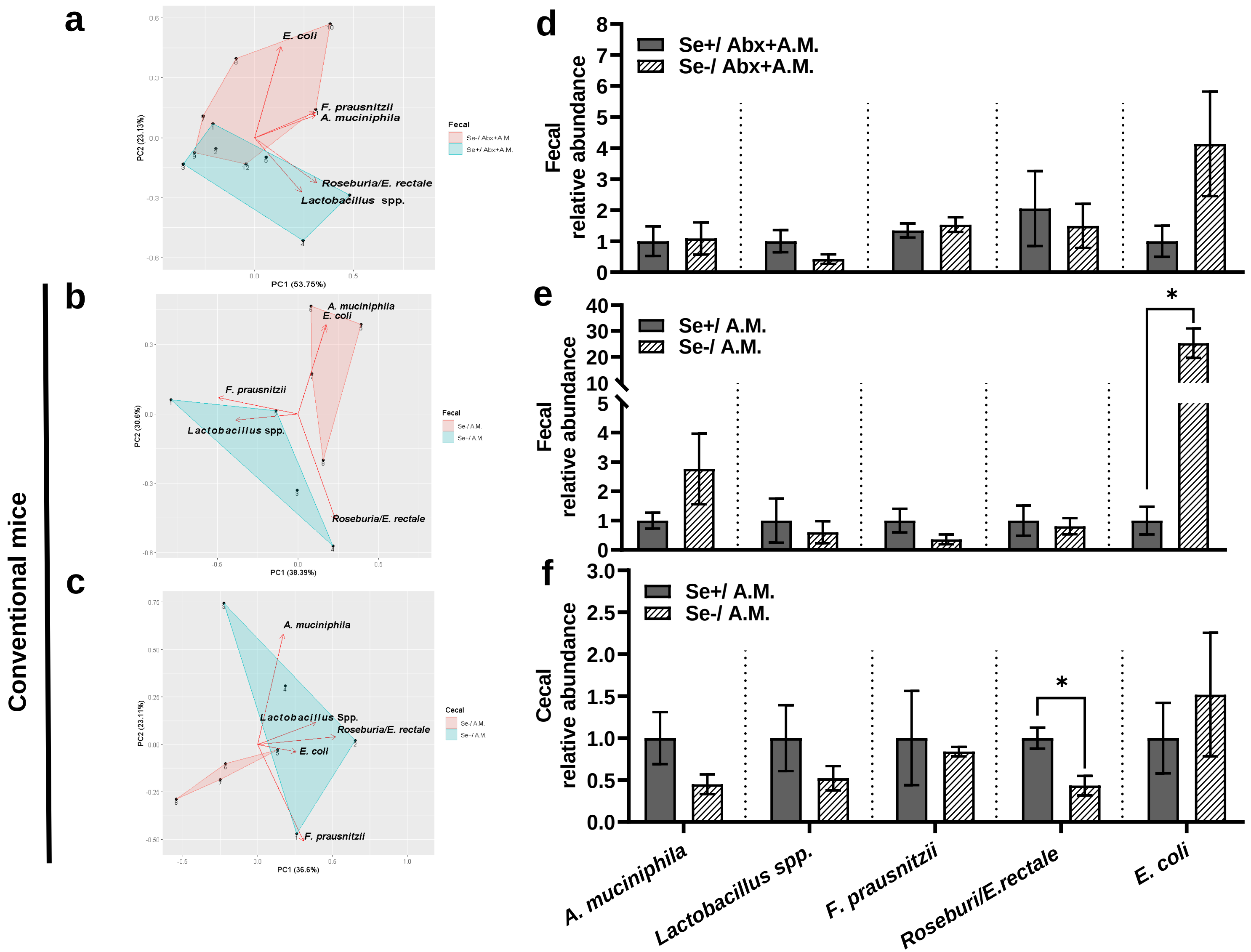

Figure S13. Visualization through principal component analysis (**a-c**) according to the $2^{-\Delta\Delta CT}$ method-quantified, qPCR-analyzed relative abundance of the 5 bacteria in feces of antibiotics-treated mice (**d**) and fecal and cecal droppings of conventional mice (**e**,**f**). See Figure 3**a** and 4**a** for study designs. The results are shown as pairwise comparisons of Se+ vs. Se- under the Abx+A.M. or A.M. condition. The most critical pairwise comparisons (Se- vs. Se+; Se- vs. Se-/A.M.) are shown in Figure 6. The arrows for each variable (bacterium) indicate the correlations with the principal components and show the direction of increasing values for each bacterium. Values (means ± SEM, n = 6-7) with a bracket differ (*$P \leq 0.05$ or indicated otherwise). Abx, antibiotics pre-treatment; A.M., *A. muciniphila* oral gavage; Se+, Se-adequate diet; Se-, Se-deficient diet.
