# Supplementary material for: Dietary Selenium Deficiency Accelerates the Onset of Aging‐Related Gut Microbial Changes in Aged Telomere‐Humanized Mice, With Akkermansia muciniphila Being the Most Prominent and Alleviating Selenium Deficiency‐Induced Type 2 Diabetes
Source: Aging Cell. 2025 Jun 20;24(8):e70130. doi: 10.1111/acel.70130 (PMC12341817; doi:10.1111/acel.70130)
Supplement: Supplementary file 2 — Appendix S2. [file ACEL-24-e70130-s001.docx]

Table S1 Primer sequences^1^

| Gene target | Sequence (5'-3') | References |
| --- | --- | --- |
| Selenoprotein |  |  |
| *Actin* | F: 5'-CTCTATGCCAACACAGTGCTGTCTGG-3' |  |
|  | R: 5'-CGTACTCCTGCTTGCTGATCCACATC-3' |  |
| *Gpx1* | F: 5'-GAGATGAACGATCTGCAGAAGCGTCTG-3' |  |
|  | R: 5'-CCTTCTCACCATTCACTTCGCACTTCTC-3' |  |
| *Gpx4* | F: 5'-ATGCACGAATTCTCAGCCAAGGACATC-3' |  |
|  | R: 5'-GCCAGGATTCGTAAACCACACTCAGC^)^-3' |  |
| *Selenoh* | F: 5'-AGACGGTGGACAAGCGCGAGAAACTG-3' |  |
|  | R: 5'-CTGTTGTCCGAGCGCAGCAGCGTCAC-3' |  |
| *Selenop* | F: 5'-CTGATACTTGTGTCTTCTGCAGGCATCC-3' |  |
|  | R: 5'-CTGTACACTGCGATGTGTTCTGACACC-3' |  |
| *Selenow* | F: 5'- TTGAGGTCCTTGTTGCTTGTGGGTCG-3' |  |
|  | R: 5'- TTCCCGGCTACTGTCACTTCAAAGAACC-3' |  |
| Barrier function |  |  |
| *Cldn3* | F: 5'-TCATCGGCAGCAGCATCATCAC-3' R: 5'-ACGATGGTGATCTTGGCCTTGG-3' | (Plovier et al., 2017) |
| *Cnr1* | F: 5'-CTGATGTTCTGGATCGGAGTC-3' R: 5'-TCTGAGGTGTGAATGATGATGC-3' | (Plovier et al., 2017) |
| *IL-6* | F: 5'-GCTACCAAACTGGATATAATCAGGA-3' R: 5'-CCAGGTAGCTATGGTACTCCAGAA-3' | (Jeffery et al., 2017) |
| TNF-α | F: 5'-GGCAGGTCTACTTTGGAGTCATTGC-3' R: 5'-ACATTCGAGGCTCCAGTGAATTCGG-3' | (Autenrieth et al., 1997) |
| *Ocln* | F: 5'-ATGTCCGGCCGATGCTCTC-3' R: 5'-TTTGGCTGCTCTTGGGTCTGTAT-3' | (Plovier et al., 2017) |
| *RPL-19* | F: 5'-GAAGGTCAAAGGGAATGTGTTCA-3' R: 5'-CCTTGTCTGCCTTCAGCTTGT-3' | (Plovier et al., 2017) |
| *ZO-1* | F: 5'-ACTCCCACTTCCCCAAAAAC-3' | (Hwang et al., 2013) |
|  | R: 5'-CCACAGCTGAAGGACTCACA-3' |  |
| Gut microbiota |  |  |
| *Akkermansia muciniphila* | F: 5'-CAGCACGTGAAGGTGGGGAC-3' R: 5'-CCTTGCGGTTGGCTTCAGAT-3' | (Collado et al., 2007) |
| *Escherichia coli* | F: 5'-GGCCTTCGGGTTGTAAAGTA-3' R: 5'-AGACTCAAGCTTGCCAGTATC-3' | (Wang et al., 1996) |
| *Faecalibacterium prausnitzii* | F: 5'-AGATGGCCTCGCGTCCGA-3' R: 5'-CCGAAGACCTTCTTCCTCC-3' | (Wang et al., 1996) |
| *Lactobacillus* spp. | F: 5'-TGGAACAGRTGCTAATACCG-3' R: 5'-GTCCATTGTGGAAGATTCCC-3' | (Byun et al., 2004) |
| *Roseburia spp. and E. rectale* | F: 5'-GCGGTRCGGCAAGTCTGA-3' R: 5'-CCTCCGACACTCTAGTMCGAC-3' | (Ramirez-Farias et al., 2009) |
|  |  |  |
| Table S1 Continued |  |  |
| *Ruminococcus torques* | F: 5'-TGCTTAACTGATCTTCTTCGGA-3' R: 5'-CGGTATTAGCAGTCATTTCTG-3' | (Kassinen et al., 2007) |
| *Universal 16S rRNA (V4 region)* | F: 5'-GCCAGCAGCCGCGGTAA-3' R: 5'-GACTACCAGGGTATCTAAT-3' |  |

^1^Abbreviations used: Cldn3, claudin 3; Cnr1, cannabinoid Receptor 1; GPX1, glutathione peroxidase-1; GPX4, glutathione peroxidase-4; IL-6, interleukin 6; SELENOH, selenoprotein H; SELENOP, selenoprotein P; SELENOW, selenoprotein W; TNF-α, tumor necrosis factor α; Ocln, occludin; RPL-19, ribosomal protein L19; ZO-1, zonula occludens-1.

Table S2 Antibodies used for immunoblotting^1^

| Antibodies | Dilution | Catalog # | Company |
| --- | --- | --- | --- |
| ***Primary antibodies*** | | | |
| AKT | 1:2000 | 9272 | Cell signaling technology, Boston, MA |
| Phospho-AKT Ser-473 | 1:1000 | 9271 | Cell signaling technology, Boston, MA |
| Phospho-AKT Thr-308 | 1:1000 | 9275 | Cell signaling technology, Boston, MA |
| GPX1 | 1:2000 | GTX116040 | GeneTex, Irvine, CA |
| GPX3 | 1:2000 | AF4199 | R&D System, Minneapolis, MN |
| SELENOH | 1:1000 | ab151023 | Abcam, Cambridge, UK |
| SELENOP | 1:1000 | GTX63138 | GeneTex, Irvine, CA |
| SELENOW | 1:1000 | NBP1-49599 | Novus Biologicals, Littleton, CO |
| β-tubulin | 1:5000 | sc-55529 | Santa Cruz Biotechnology, Santa Cruz, CA |
| Albumin | 1:5000 | 4929 | Cell signaling technology, Boston, MA |
|  |  |  |  |
| ***Secondary antibodies*** | | | |
| Anti-mouse | 1:5000 | 7076 | Cell signaling technology, Boston, MA |
| Anti-rabbit | 1:5000 | 7074 | Cell signaling technology, Boston, MA |
| Anti-goat | 1:5000 | sc-2378 | Santa Cruz Biotechnology, Santa Cruz, CA |

^1^Abbreviations used: AKT, thymoma viral proto-oncogene (mice) or AKT serine/threonine kinase (humans); GPX1, glutathione peroxidase-1; GPX3, glutathione peroxidase-3; SELENOH, selenoprotein H; SELENOP, selenoprotein P; SELENOW, selenoprotein W.

**References:**

Autenrieth, I. B., Bucheler, N., Bohn, E., Heinze, G., & Horak, I. (1997). Cytokine mRNA expression in intestinal tissue of interleukin-2 deficient mice with bowel inflammation. *Gut, 41*(6), 793-800. doi:10.1136/gut.41.6.793

Byun, R., Nadkarni, M. A., Chhour, K. L., Martin, F. E., Jacques, N. A., & Hunter, N. (2004). Quantitative analysis of diverse Lactobacillus species present in advanced dental caries. *Journal of clinical microbiology, 42*(7), 3128-3136. doi:10.1128/jcm.42.7.3128-3136.2004

Collado, M. C., Derrien, M., Isolauri, E., de Vos, W. M., & Salminen, S. (2007). Intestinal integrity and Akkermansia muciniphila, a mucin-degrading member of the intestinal microbiota present in infants, adults, and the elderly. *Appl Environ Microbiol, 73*(23), 7767-7770. doi:10.1128/aem.01477-07

Hwang, I., An, B. S., Yang, H., Kang, H. S., Jung, E. M., & Jeung, E. B. (2013). Tissue-specific expression of occludin, zona occludens-1, and junction adhesion molecule A in the duodenum, ileum, colon, kidney, liver, lung, brain, and skeletal muscle of C57BL mice. *J Physiol Pharmacol, 64*(1), 11-18.

Jeffery, V., Goldson, A. J., Dainty, J. R., Chieppa, M., & Sobolewski, A. (2017). IL-6 Signaling Regulates Small Intestinal Crypt Homeostasis. *J Immunol, 199*(1), 304-311. doi:10.4049/jimmunol.1600960

Kassinen, A., Krogius-Kurikka, L., Mäkivuokko, H., Rinttilä, T., Paulin, L., Corander, J., . . . Palva, A. (2007). The fecal microbiota of irritable bowel syndrome patients differs significantly from that of healthy subjects. *Gastroenterology, 133*(1), 24-33. doi:10.1053/j.gastro.2007.04.005

Plovier, H., Everard, A., Druart, C., Depommier, C., Van Hul, M., Geurts, L., . . . Cani, P. D. (2017). A purified membrane protein from Akkermansia muciniphila or the pasteurized bacterium improves metabolism in obese and diabetic mice. *Nat Med, 23*(1), 107-113. doi:10.1038/nm.4236

Ramirez-Farias, C., Slezak, K., Fuller, Z., Duncan, A., Holtrop, G., & Louis, P. (2009). Effect of inulin on the human gut microbiota: stimulation of Bifidobacterium adolescentis and Faecalibacterium prausnitzii. *Br J Nutr, 101*(4), 541-550. doi:10.1017/s0007114508019880

Wang, R. F., Cao, W. W., & Cerniglia, C. E. (1996). Phylogenetic analysis of Fusobacterium prausnitzii based upon the 16S rRNA gene sequence and PCR confirmation. *Int J Syst Bacteriol, 46*(1), 341-343. doi:10.1099/00207713-46-1-341
